# Self‐Assembly Hypoxic and ROS Dual Response Nano Prodrug as a New Therapeutic Approach for Glaucoma Treatments

**DOI:** 10.1002/advs.202407043

**Published:** 2024-09-04

**Authors:** Xuezhi Zhou, Rong Rong, Ganghao Liang, Yukun Wu, Chun Xu, Haihua Xiao, Dan Ji, Xiaobo Xia

**Affiliations:** ^1^ Department of Ophthalmology Xiangya Hospital, Central South University Changsha Hunan 410008 P. R. China; ^2^ Hunan Key Laboratory of Ophthalmology Changsha Hunan 410008 P. R. China; ^3^ National Clinical Research Center for Geriatric Diseases Central South University Changsha Hunan 410008 P. R. China; ^4^ Beijing National Laboratory for Molecular Sciences State Key Laboratory of Polymer Physics Institute of Chemistry Chinese Academy of Sciences Beijing 100190 P. R. China; ^5^ University of Chinese Academy of Sciences Beijing 100049 P. R. China; ^6^ School of Dentistry The University of Queensland Brisbane 4006 Australia

**Keywords:** apoptosis, axonal damage, glaucoma, HOLN‐NPs, retina ganglion cells

## Abstract

Glaucoma is an irreversible blinding eye disease characterized by retinal ganglion cell (RGC) death.Previous studies have demonstrated that protecting mitochondria and activating the CaMKII/CREB signaling pathway can effectively protect RGC and axon. However, currently treatments are often unsatisfactory, and the pathogenesis of glaucoma requires further elucidation. In this study, a ROS‐responsive dual drug conjugate (OLN monomer) is first designed that simultaneously bonds nicotinamide and oleic acid. The conjugate self‐assembled into nanoparticles (uhOLN‐NPs) through the aggregation of multiple micelles and possesses ROS scavenging capability. Then, a polymer with a hypoxic response function is designed, which encapsulates uhOLN‐NPs to form nanoparticles with hypoxic and ROS responses (HOLN‐NPs). Under hypoxia in RGCs, the azo bond of HOLN‐NPs breaks and releases uhOLN‐NPs. Meanwhile, under high ROS conditions, the thioketone bond broke, leading to the dissociation of nano‐prodrug. The released nicotinamide and oleic acid co‐scavenge ROS and activate the CaMKII/CREB pathway, protecting mitochondria in RGCs. HOLN‐NPs exhibit a significantly superior protective effect on R28 cells in glutamate models of glaucoma. The accumulation of HOLN‐NPs in retinal RGCs lead to significant inhibition of RGC apoptosis and axonal damage in vivo. Notably, HOLN‐NPs provide a new therapeutic approach for patients with neurodegenerative disease.

## Graphical abstract

A dual hypoxia and ROS‐responsive nano drug system for Nico and OA (HOLN‐NPs) can be injected into the eye to protect RGCs. HOLN‐NPs show promising potential in protecting RGCs from apoptosis and axonal damage, providing a new therapeutic approach for patients with neurodegenerative disease.

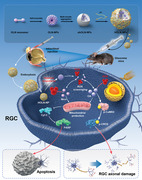

## 1 Introduction

Glaucoma is a progressive optic neurodegenerative disease and the leading cause of irreversible blindness, affecting ≈80 million people worldwide.^[^ ^1^ ^]^ Retinal ganglion cell (RGC) death and axon injury are pathophysiological signs of glaucoma.^[^ ^2^ ^]^ RGCs are important neurons that carry visual information from the retina to the brain, and their damage and death result in severe visual impairment.^[^ ^3^ ^]^ Currently, the treatment approach for glaucoma primarily revolves around managing intraocular pressure through diverse drugs, laser treatment, or surgical interventions to slow its progression.^[^ ^4^ ^]^ However, the efficacy of these treatments remains limited, and the persistent decline and death of the RGC eventually lead to blindness in some glaucoma patients.^[^ ^5^ ^]^ In parallel, attempts have been made to administer neuroprotective drugs orally or intravenously. However, this approach has several challenges, such as the limited amount of drug that reaches the eye due to body metabolism and the blood‐eye barrier.^[^ ^6^ ^]^ In addition, frequent oral and intravenous administration can substantially reduce patient compliance. Therefore, it is important to further study the pathogenesis of glaucoma, seek new effective treatment methods and solutions, and enhance the protection of RGCs in glaucoma.

Although the cause of glaucoma remains unknown, oxidative stress‐induced damage is thought to be a critical factor in RGC death.^[^ ^7^ ^]^ The excessive reactive oxygen species (ROS) accumulation not only triggers the opening of mitochondrial bilayer membrane pores but also leads to the release of calcium ions, cytochrome C (Cyt C), apoptosis‐inducing factor (AIF), subsequently activating caspase 3/6/7 through caspase 9.^[^ ^8^ ^]^ In addition, the electron transport chain of mitochondria can be uncoupled, leading to down‐regulation of Adenosine triphosphate (ATP) production, up‐regulation of pro‐apoptotic protein BCL2‐Associated X (Bax) expression, and ultimately, breakdown of mitochondrial outer membrane and cell apoptosis.^[^ ^9^ ^]^ Therefore, effective clearance of ROS and protection of mitochondria may be crucial strategies for neuroprotection in glaucoma.

Currently, two primary protective strategies are employed for RGCs in glaucoma. The first strategy is to use antioxidants to remove ROS from RGCs.^[^ ^10^, ^11^ ^]^ However, antioxidants are limited in their effectiveness due to their low efficiency of entry into RGCs, short and uncontrollable action times, and potential side effects. Moreover, current ROS‐consuming materials such as selenium/telluride‐containing polymers, diselenide/telluride‐containing polymers, and polyoxalates face challenges; they include difficult synthesis, poor controllability, poor degradability, potential toxicity, single action mechanism, and insufficient final efficacy.^[^ ^12^, ^13^, ^14^, ^15^ ^]^ The second strategy is to develop small‐molecule drugs, protein drugs, and gene therapy to protect RGCs.^[^ ^2^, ^10^, ^16^, ^17^ ^]^ Small molecule drugs or protein drugs have the problems of poor solubility, easy degradation, and short duration of action. Gene therapies often use viral vectors, so there are biological safety problems. Therefore, effective treatment of glaucoma by protecting RGCs remains a crucial challenge requiring careful and innovative approaches.

Nicotinamide (Nico) is an amide form of vitamin B3 whose therapeutic potential has been demonstrated for multiple diseases, including skin diseases, diabetes, cancer metastases, cerebral ischemia, multiple sclerosis, Alzheimer's disease, viral and microbial infections, inflammatory diseases, both animal studies and clinical trials.^[^ ^18^, ^19^, ^20^, ^21^ ^]^ Nico can not only inhibit the formation of abnormal mitochondria by consuming intracellular ROS but also protect RGCs from damage by down‐regulating Cyt C levels and inhibiting the expression of Poly ADP‐ribose polymerase (PARP) and Phosphorylated histone H2AX (γ‐H2AX).^[^ ^22^, ^23^ ^]^ Oleic acid (OA) is a fatty acid found naturally in different types of plant and animal lipids.^[^ ^24^ ^]^ It has strong antioxidant activity and is a Calcium Calmodulin Dependent Protein Kinase II (CaMKII) agonist, which can protect neurons by clearing intracellular ROS and activating the CaMKII/CREB signaling pathway.^[^ ^25^, ^26^ ^]^ Recent studies have indicated that activation of the CaMKII/CREB signaling pathway not only effectively protects RGC cell bodies and axons from damage but also protects remote projection of RGC axons in vivo, thus protecting visual functions from the retina to the visual cortex.^[^ ^17^ ^]^ Nico is water soluble, and its action time is short and uncontrollable. The water solubility of OA is poor, and its efficacy is affected. Delivering both Nico and OA into RGC to clear ROS while protecting mitochondria and activating the CaMKII/CREB signaling pathway is challenging.

Herein, we designed a dual hypoxia and ROS‐responsive nano drug system for Nico and OA that can be injected into the eye to protect RGCs. The nano drug (HNLO‐NPs) is created by self‐assembling a polymer featuring a thioketone bond, enabling ROS response and clearance functions. Then it is enveloped by another polymer with hypoxia‐responsive properties. We assessed HNLO‐NPs in three mouse models (mice^NMDA^, mice^ONC^, and mice^IR^) replicating glutamate excitatory toxicity, optic nerve crush and ischemia reperfusion to mimic the pathophysiological characteristics of glaucoma RGCs injury. In the hypoxia environment of RGCs, the azo bond of HOLN‐NPs broke and released uhOLN‐NPs in vivo. Moreover, excessive ROS in RGCs trigger the thioketone bond breakage in uhOLN‐NPs, leading to polymer dissociation, ROS consumption, and the release of Nico and OA. Nico release effectively protected mitochondria and reduced intracellular ROS, thus inhibiting RGCs apoptosis. Simultaneously, OA activated the CaMKII/CREB signaling pathway, preventing RGC apoptosis and axonal damage while depleting intracellular ROS. In conclusion, the bonding of Nico and OA via the thioketone bond formed ROS‐responsive uhOLN‐NPs through self‐assembly and multi‐micellar aggregation. Additionally, HOLN‐NPs emerged after coating with the hypoxia‐responsive polymer P1, offering mitochondrial protection and CaMKII/CREB pathway activation. Our approach provides a new strategy for neuroprotection.

## 2 Results and Discussion

### 2.1 Preparation and Characterization of HOLN‐NPs

**Scheme 1:**
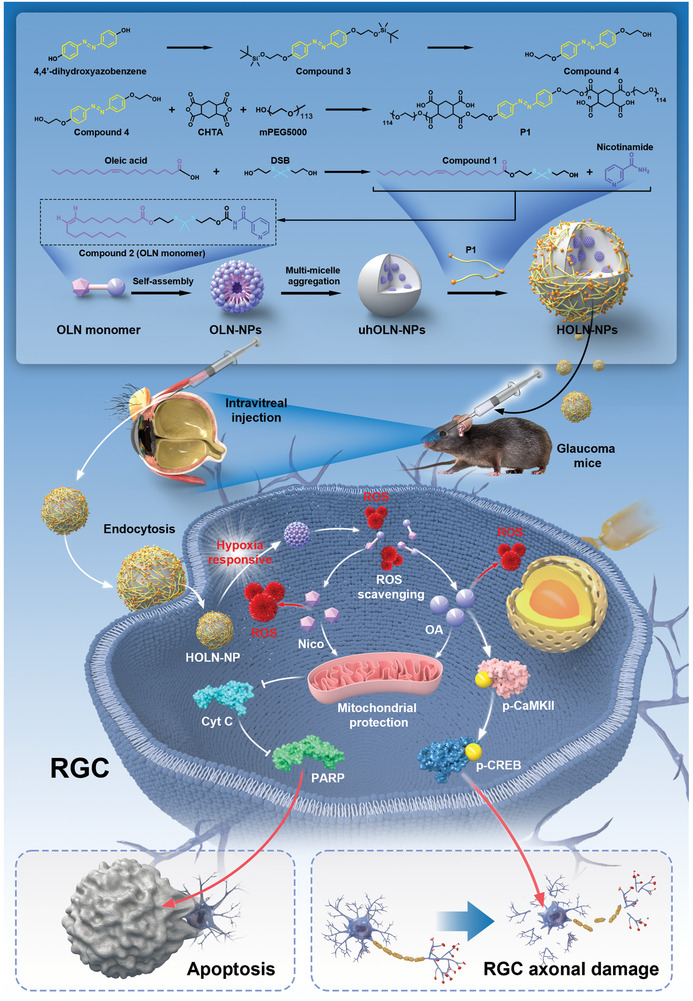
Schematic illustration of HOLN‐NPs that inhibit apoptosis and axonal damage for glaucoma RGC. First, during the damage process of glaucoma disease, HOLN‐NPs were activated by hypoxia, and azo bonds were opened to release uhOLN‐NPs in response. Second, under the stimulation of ROS, the thioketal bond of OLN‐NPs was open, and Nico and OA were released while scavenging ROS. Third, Nico inhibited RGC apoptosis through the Cyt C/PARP pathway. Finally, OA acted as a p‐CaMKII agonist to protect RGC from apoptosis and axonal damage by activating the CaMKII/CREB pathway.

To deliver Nico and OA into the RGC more safely and efficiently, the polymer OLN monomer was synthesized by bonding them through a thioketone bond (Figures S1–S3, Supporting Information). The thioketal bond of OLN monomer could be broken by ROS, thereby scavenging ROS. After forming OLN‐NPs by self‐assembly, uhOLN‐NPs were formed by multi‐micellar aggregation. HOLN‐NPs were formed when P1 was coated with uhOLN‐NPs in response to hypoxia (**Scheme** **1** and Figures S4–S6, Supporting Information).

**Figure 1:**
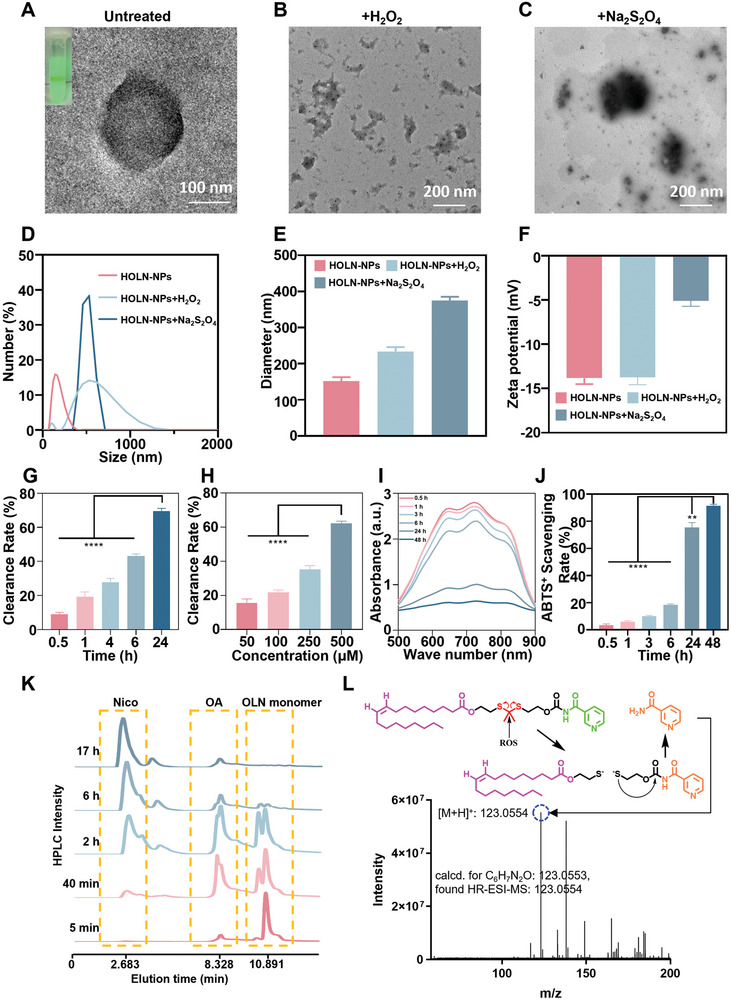
Characterization, hypoxic responsiveness, and scavenging ROS of HOLN‐NPs. A–C) Representative TEM images of HOLN‐NPs, HOLN‐NPs + H_2_O_2_, and HOLN‐NPs + Na_2_S_2_O_4_. D,E) Hydrodynamic diameters of HOLN‐NPs, HOLN‐NPs + H_2_O_2_, and HOLN‐NPs + Na_2_S_2_O_4_ by DLS. F) Zeta potential of HOLN‐NPs, HOLN‐NPs + H_2_O_2_, and HOLN‐NPs + Na_2_S_2_O_4_ by DLS. G) Scavenging H_2_O_2_ by HOLN‐NPs at various time points. H) Scavenging H_2_O_2_ by HOLN‐NPs at different concentrations at 6 h. I,J) Scavenging ∙ABTS^+^ by HOLN‐NPs at various time points. K) HPLC analysis of Nico and OA release triggered by 1 mm H_2_O_2_ at different time points. L) LTQ‐MS result of Nico release from OLN monomer in the presence of 1 mm H_2_O_2_. Statistical significance was calculated via one‐way ANOVA analysis. **p < 0.01, ****p < 0.0001.

HOLN‐NPs have good water solubility due to the use of amphiphilic polymers P1 encapsulating the prodrugs, which increases water solubility. Transmission electron microscopy (TEM) showed that HOLN‐NPs had a uniform spherical nanostructure (**Figure** **1**A). After incubation with H_2_O_2_ and Na_2_S_2_O_4_, HOLN‐NPs had an aggregated, swollen morphology (Figure 1B,C). The particle size of HOLN‐NPs was 151.5 nm with a zeta potential at −27.6 mV, and a polydispersity index (PDI) at 0.18 by Dynamic Light Scattering (DLS) (Figure 1D–F). These results confirmed the successful preparation of HOLN‐NPs. To evaluate the ROS scavenging ability, HOLN‐NPs were treated with H_2_O_2_. The results showed that the particle size of HOLN‐NPs increased from 151.5 to 233.1 nm, the zeta potential increased from −27.63 to −27.49 mV, and the PDI increased from 0.18 to 0.46. To evaluate the hypoxia response ability of HOLN‐NPs, they were treated with Na_2_S_2_O_4_. The particle size of HOLN‐NPs increased to 524.2 nm, the zeta potential increased to −10.2 mV, and the PDI increased to 0.94 (Figure 1D–F). The larger particle size and larger PDI of HOLN‐NPs treated with H_2_O_2_ and Na_2_S_2_O_4_ indicated that they have dual responsiveness to hypoxia and ROS. However, upon H_2_O_2_ and Na_2_S_2_O_4_ treatment, the particle size of the HOLN‐NPs significantly increased, confirming that HOLN‐NPs could be dissociated. Both particle size and zeta potential changed significantly when both stimuli were applied simultaneously. The average particle size of the nanoparticles decreased to 18.4 nm and the zeta potential increased to −2.0 mV.

Subsequently, H_2_O_2_ and 2, 2′‐azino‐bis (3‐ethylbenzothiazoline‐6‐sulfonic acid) diammonium salt (∙ABTS^+^) were adopted to incubate HOLN‐NPs to further evaluate their ROS scavenging ability. HOLN‐NPs had a time‐dependent effect on H_2_O_2_ scavenging. Approximately 70% of H_2_O_2_ was consumed after incubating HOLN‐NPs with H_2_O_2_ for 24 h (Figure 1G). The effect of H_2_O_2_ scavenging was dose‐dependent (Figure 1H). Similarly, ≈90% of ∙ABTS^+^ were consumed after incubating the HOLN‐NPs with ∙ABTS^+^ for 48 h (Figure 1I), which was also in a time‐dependent manner (Figure 1J). Taken together, the above results confirmed that HOLN‐NPs have ROS responsiveness and the ability to scavenge ROS, providing a theoretical basis for protecting RGC from oxidative stress damage.

The OLN monomer was expected to release Nico and OA under excessive ROS conditions. To determine the release of Nico and OA, the OLN monomer was incubated in a 1 mm H_2_O_2_ solution at various time points to simulate high ROS conditions. The reaction was then monitored by HPLC. With prolonged incubation time with H_2_O_2_, the peak of OLN monomer at 5 min gradually decreases, as shown in Figure 1K. In contrast, two new peaks representing Nico and OA appeared and increased. As OA also consumed H_2_O_2_, a large amount of OA was released when the OLN monomer was degraded in the early stage. Due to the extension of time, the generated OA also decreased gradually due to the consumption of H_2_O_2_ (Figure 1K). A linear ion trap mass spectrometer (LTQ‐MS) was employed to identify the degradation products. The peaks at m/z 123.0554 belonging to Nico were observed after the OLN monomer was incubated with H_2_O_2_ (Figure 1L). These results suggested the H_2_O_2_ triggered the dissociation of the OLN monomer and the controlled release of Nico and OA. The degradation mechanism of the OLN monomer could be divided into three steps: the reduction of the thioketal bond, a spontaneous cascade of dissociation reactions, and the release of Nico and OA.

### 2.2 Intracellular Uptake, ROS Scavenging Ability, and Inhibition of R28^Glu^ Cell Apoptosis of HOLN‐NPs

**Figure 2:**
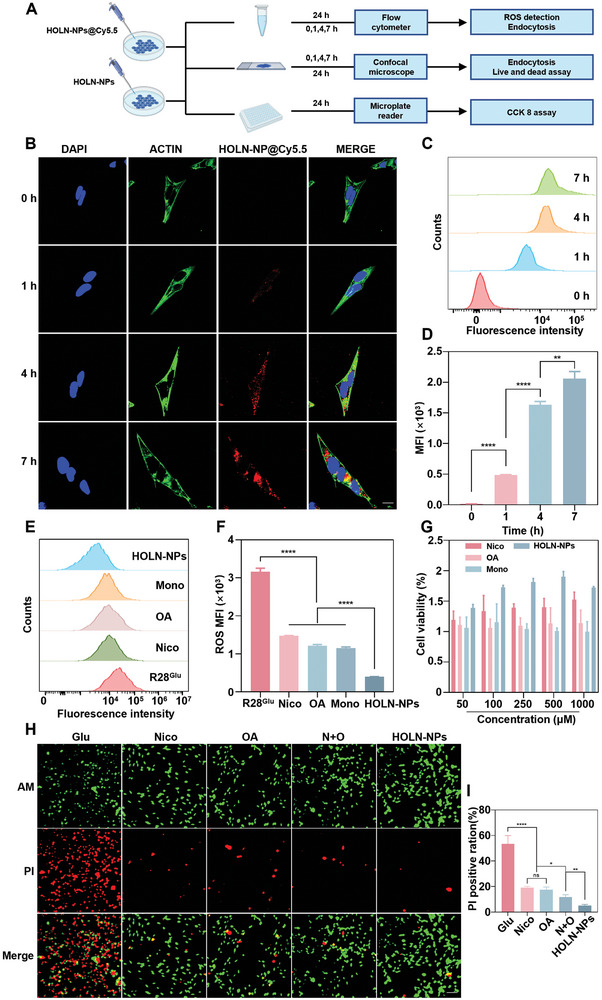
Intracellular uptake, ROS scavenging, and apoptosis inhibition of HOLN‐NPs in vitro. A) Schematic illustration of the intracellular uptake, the subsequent ROS scavenging, and apoptosis inhibition of HOLN‐NPs in R28 cells. B) Representative CLSM images of R28 cells treated with HOLN‐NPs@Cy5.5 at 0, 1, 4, and 7 h, respectively. The cell nucleus was stained by DAPI (blue). The red fluorescence came from Cy5.5 (red). The cell skeleton was stained by Actin‐Tracker Green‐488 (green) respectively. Scale bar = 10 µm. C,D) Flow cytometric profiles and corresponding semi‐quantification of intracellular uptake of HOLN‐NPs@Cy5.5 at 0, 1, 4, and 7 h, respectively. E,F) Detection of ROS by DCFH‐DA in R28^Glu^ cells treated with Nico, OA, monomer, and HOLN‐NPs, respectively, by flow cytometry. G) In vitro cell viability of R28^Glu^ cells by Nico, OA, monomer, and HOLN‐NPs at different concentrations by CCK‐8. H,I) The representative CLSM images of R28^Glu^ were stained with Calcein‐AM (green) and PI (red) after treatment with Nico, OA, N + O, and HOLN‐NPs for 24 h. Scale bar = 50 µm. Data are presented as the mean ± SD (*n* = 3–5). Statistical significance was calculated via one‐way ANOVA analysis. *p < 0.05, **p < 0.01, ***p < 0.001, and ****p < 0.0001.

To study the uptake of HOLN‐NPs, R28 cells were treated with Cy5.5‐labeled nanoparticles (HOLN‐NPs@Cy5.5), which were then observed under CLSM (**Figure** **2**A,B). Results showed that the red fluorescence of HOLN‐NPs@Cy5.5 could localize in the cytoplasm of R28 cells, which revealed the accumulation of HOLN‐NPs@Cy5.5 in cells. In addition, the uptake of HOLN‐NPs@Cy5.5 was further studied by flow cytometry (Figure 2C,D). The fluorescence intensity of HOLN‐NPs@Cy5.5 uptake by R28 cells at 7 h was nearly four times as much as that at 1 h. We labeled the lysosomes with Lamp 1 antibody and performed endocytosis experiments with HOLN‐NP@Cy5.5 at 4 and 7 h. The experimental results showed that Cy5.5 (red) and Lamp 1 (green) have significant co‐localization in R28 cells, which indicates that there is no significant lysosomal escape of nanoparticles (Figure S7, Supporting Information).

We then tested HOLN‐NPs‐mediated intracellular ROS scavenging and mitigated hypoxia using ROS/hypoxia detection probes. We constructed a glutamate cytotoxicity model, named R28^Glu^, to simulate the RGC damage model of glaucoma in vitro. To investigate whether HOLN‐NPs could scavenge ROS in vitro, the intracellular ROS level in R28^Glu^ treated with HOLN‐NPs was studied by a fluorescent probe DCFH‐DA. Hypoxyprobe‐1 was used to reflect a hypoxic gradient in cells. The untreated R28 cells were set as negative control (R28^Norm^), and R28^Glu^ cells were set as positive controls. After incubation with HOLN‐NPs in different groups, the levels of ROS and hypoxia in R28 cells were investigated by CLSM (Figures S8 and S9, Supporting Information). The stronger green fluorescence signal of ROS generation and the red signal of hypoxia production were observed in R28^Glu^ cells. However, the R28^Norm^ group and R28^Glu^ cells treated with HOLN‐NPs exhibited minor green and red fluorescence, demonstrating ROS scavenging and hypoxia alleviation under HOLN‐NPs treatment. Further quantitative measurements of intracellular ROS reduction were performed by flow cytometry (Figure 2E,F). The ROS level of R28^Glu^ cells treated with HOLN‐NPs was ≈12.6% of the R28^Glu^ group, respectively, which was much lower than those of the Nico group, OA group, and Nico + OA group. These results indicated that HOLN‐NPs had the strongest ability to scavenge ROS in R28^Glu^ cells and could effectively relieve the hypoxic state of the R28^Glu^ cells.

Next, the in vitro cell viability of Nico, OA, monomer, and HOLN‐NPs on R28^Glu^ and A Oxygen Glucose Deprivation cell model (R28^OGD^)cells was investigated through Cell Counting Kit‐8 (CCK‐8) assay. The results showed that as the concentration of Nico increased, the proliferation activity of R28^Glu^ cells gradually increased (Figures S10 and S11, Supporting Information). After 24 h of treatment, the optimal protective concentration of Nico for R28^Glu^ was 10 µm. However, at higher concentrations (100 and 500 mm), Nico exhibited significant toxicity to R28^Glu^. The optimal protective concentration of OA on R28^Glu^ cells was 1 µm. At a concentration of 1 mm, OA caused significant toxicity to R28^Glu^ cells (Figures S12 and S13, Supporting Information). Subsequently, we used the CCK‐8 assay to evaluate the effect of HOLN‐NPs on the viability of R28^Glu^ cells (Figure 2G). The results showed that the protective effect of HOLN‐NPs on R28^Glu^ gradually increases with increasing concentration. When its concentration was 500 µm, it exhibited the optimal protective effect on R28^Glu^ cells. Its protective effect was significantly stronger than that of the Nico, OA, and Monomer groups. The most significant enhancement of R28^OGD^ cell viability was observed with HOLN‐NPs at a concentration of 500 µm (Figure S14, Supporting Information).

To further investigate the anti‐apoptosis ability of HOLN‐NPs, R28^Glu^ cells were labeled with Calcein Acetoxy Methylester (Calcein‐AM) and propidium iodide (PI) to differentiate live/dead cells. CLSM images showed that the number of R28 cells with red fluorescence (PI, cell dead) staining was the largest in the R28^Glu^ group, accounting for ≈50% of the total number of cells. The number of cells stained with red fluorescence in the Nico group, OA group, and N + O group was lower than that in the R28^Glu^ group. However, the number of cells stained with red fluorescence was the lowest in the HOLN‐NPs group, approximately one‐tenth of the number in the R28^Glu^ group (Figure 2H,I). The above‐mentioned results indicated that HOLN‐NPs were more effective in inhibiting R28^Glu^ cell death.

### 2.3 Effects of HOLN‐NPs on Mitochondrial Membrane Potential, Cell Cycle and Apoptosis

**Figure 3:**
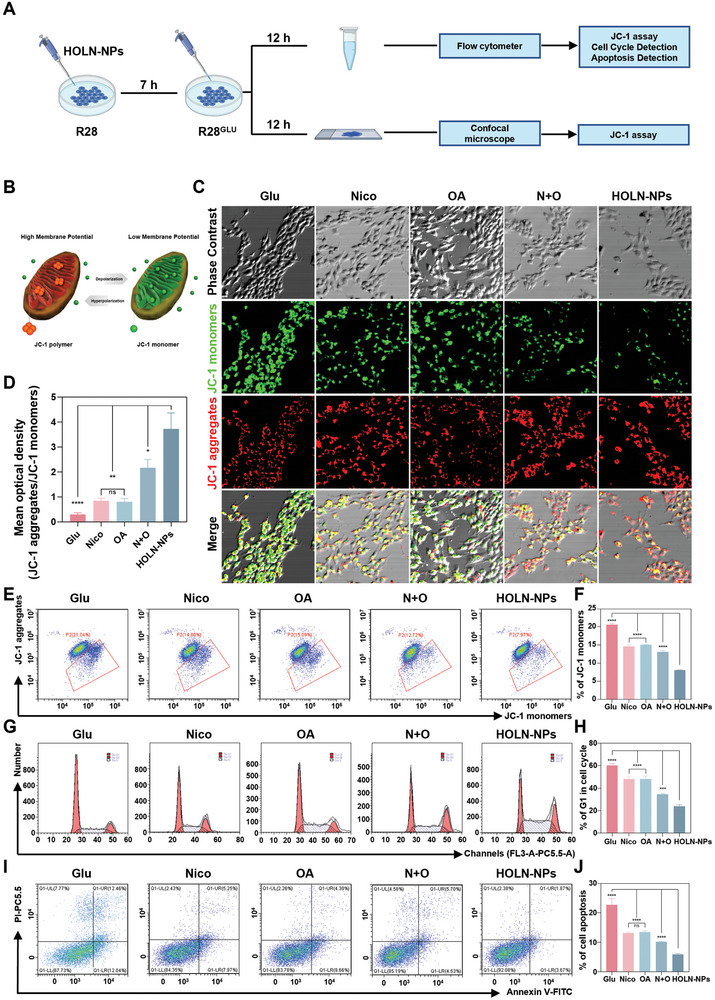
Effects of HOLN‐NPs on mitochondrial membrane potential, cell cycle and apoptosis. A) Schematic illustration of the effect on mitochondrial membrane potential, cell cycle, and apoptosis of HOLN‐NPs in R28^Glu^ cells. B–D) The representative CLSM images of R28^Glu^ were stained with a JC‐1 fluorescent probe after treatment with Nico, OA, N + O, and HOLN‐NPs for 24 h. Scale bar = 50 µm. E,F) The Mitochondrial membrane potential of R28^Glu^ was stained with a JC‐1 fluorescent probe after treatment with Nico, OA, N + O, and HOLN‐NPs for 24 h by flow cytometry. G,H) The cell cycle of R28^Glu^ was stained with PI after treatment with Nico, OA, N + O, and HOLN‐NPs for 12 h by flow cytometry. I,J) Flow cytometric profiles (I) and corresponding semi‐quantification J) of apoptotic rates in R28^Glu^ with various treatments. Statistical significance was calculated via one‐way ANOVA analysis. *p < 0.05, **p < 0.01, ***p < 0.001, and ****p < 0.0001.

JC‐1 is a kind of ideal fluorescent probe widely used to detect mitochondrial membrane potential (∆Ψm). We evaluated the effect of HOLN‐NPs on the mitochondrial membrane potential of R28^Glu^ cells by confocal and flow cytometry (**Figure** **3**A–D). CLSM images showed that the fluorescence intensity ratio of JC‐1 aggregates to the JC‐1 monomer was ≈3.7% in R28^Glu^ cells treated with HOLN‐NPs, and ≈0.3% in untreated R28^Glu^ cells. Although the fluorescence intensity ratio was higher in R28^Glu^ cells treated with Nico, OA, and N + O than in untreated R28^Glu^ cells, it was lower than that in R28^Glu^ cells treated with HOLN‐NPs. Flow cytometry results were consistent with confocal results. The proportion of JC‐1 monomers is ≈8% in R28^Glu^ cells treated with HOLN‐NPs, and ≈20% in untreated R28^Glu^ cells (Figure 3E,F). Notably, HOLN‐NPs can significantly increase the mitochondrial membrane potential of R28^Glu^ cells and inhibit the apoptosis of R28^Glu^ cells.

Research has indicated that apoptosis of RGC is associated with cell cycle changes during the pathogenesis of glaucoma. Apoptosis is often accompanied by cell cycle arrest. Blocking the process of the cell proliferation cycle can cause apoptosis, indicating a close relationship between the cell cycle and apoptosis. As shown in Figure 3G,H, the proportion of the G0/G1 phase of R28^Glu^ cells decreased by ≈12% after Nico and OA treatment, while that of R28^Glu^ cells was reduced by ≈25% after N + O treatment. The proportion of G0/G1 phase of R28^Glu^ cells treated with HOLN‐NPs decreased by ≈36%, which indicated that HOLN‐NPs could prevent R28^Glu^ cells apoptosis by regulating the cell cycle. Finally, we evaluated the apoptosis level of R28^Glu^ cells treated with different drugs using Annexin V‐FITC and PI double staining assay by flow cytometry (Figure 3I,J). The results showed that the apoptosis rate of R28^Glu^ cells treated with HOLN‐NPs was ≈6%, significantly lower than that of other drug treatment groups, and was nearly a quarter of the apoptosis rate of the R28^Glu^ group (≈23%). The above results indicate that HOLN‐NPs could efficiently inhibit R28^Glu^ apoptosis and were more effective than Nico, OA, and N + O.

### 2.4 HOLN‐NPs Protect RGC by Activating the CaMKII/CREB Pathway

**Figure 4:**
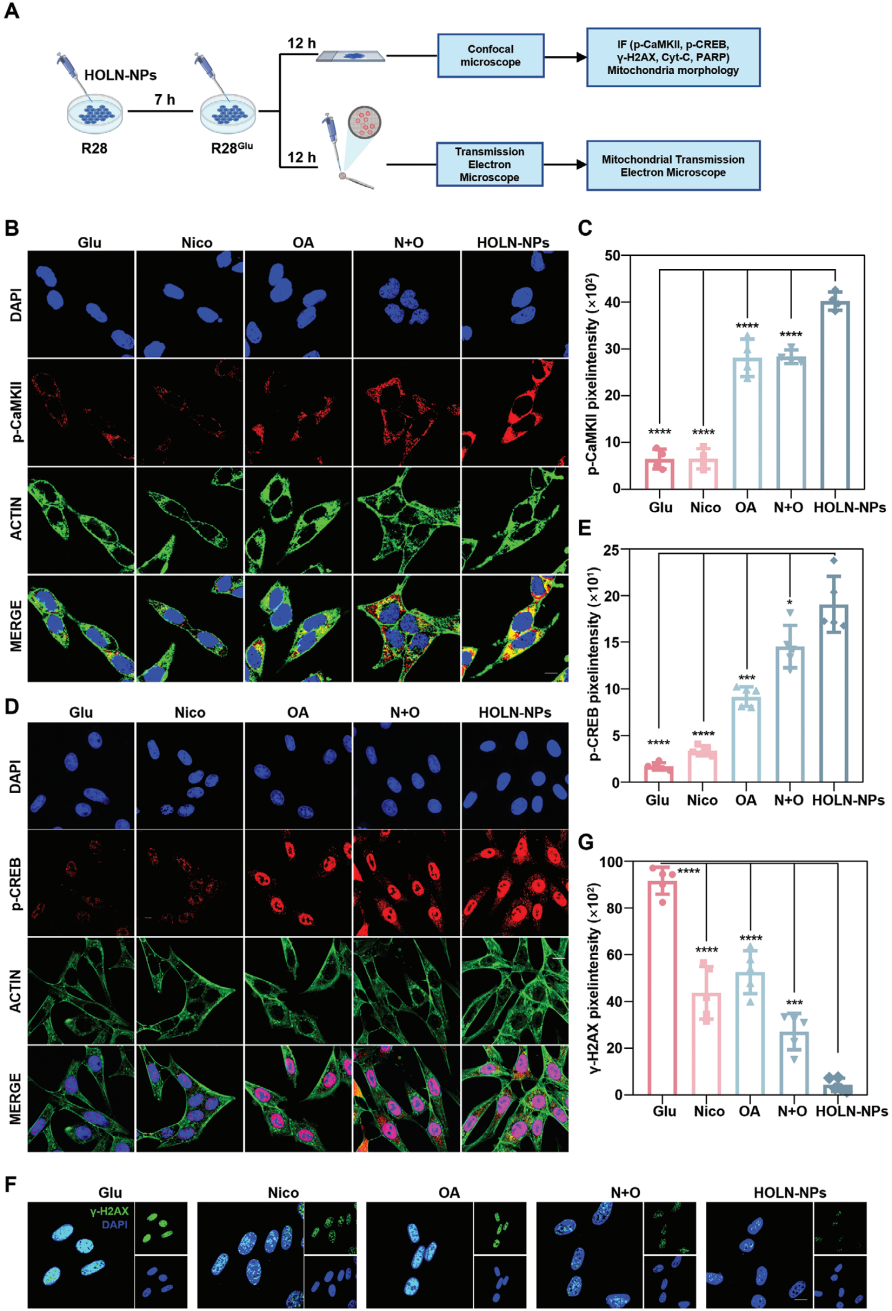
HOLN‐NPs could activate the CaMKII/CREB signal pathway. A) Schematic illustration of the effect on the p‐CaMKII/p‐CREB signal pathway of HOLN‐NPs in R28^Glu^ cells. B) The expression of p‐CaMKII in R28^Glu^ with various treatments by IF. Scale bar = 10 µm. C) The relative pixel intensity of p‐CaMKII in R28^Glu^ with various treatments. D) The expression of p‐CREB in R28^Glu^ with various treatments by IF. Scale bar = 10 µm. E) The relative pixel intensity of p‐CREB in R28^Glu^ with various treatments. F) The expression of γ‐H2AX in R28^Glu^ with various treatments by IF. Scale bar = 10 µm. G) Relative pixel intensity of γ‐H2AX in R28^Glu^ with various treatments. Data are presented as the mean ± SD (n = 5). Statistical significance was calculated via one‐way ANOVA analysis. *p < 0.05, **p < 0.01, ***p < 0.001, and ****p < 0.0001.

The activation of the CaMKII/CREB pathway is a potential therapeutic target for protecting glaucoma RGCs in the setting of glaucoma. OA is an effective p‐CaMKII agonist, and its significant neuronal protective function has been confirmed in a previous study.^[^ ^25^ ^]^ To investigate whether HOLN‐NPs protect RGCs through the activation mechanism of the CaMKII/CREB pathway, we first qualitatively and semi‐quantitatively analyzed the expression level of p‐CaMKII (red fluorescence) in R28^Glu^ cells by immunofluorescence staining (**Figure** **4**A). The results showed that R28^Glu^ cells and R28^Glu^ cells treated with Nico showed weak red fluorescence, while R28^Glu^ cells treated with OA and N + O showed significant enhancement of intracellular red fluorescence (Figure 4B,C). However, the red fluorescence intensity in the cytoplasm of R28^Glu^ cells treated with HOLN‐NPs was the strongest, and the fluorescence intensity was approximately six times that in R28^Glu^ cells, indicating that HOLN‐NPs could significantly enhance the protein expression of p‐CaMKII. Subsequently, we detected the expression of p‐CREB (red fluorescence) in cells of different treatment groups by immunofluorescence staining (Figure 4D,E). The confocal results showed that R28^Glu^ cells and R28^Glu^ cells treated with Nico showed weak red fluorescence, while the red fluorescence in R28^Glu^ cells was significantly enhanced after OA and N + O treatment. The R28^Glu^ cells treated with HOLN‐NPs showed the strongest intracellular red fluorescence, which was ≈11 times the intensity of red fluorescence in R28^Glu^ cells. Compared with other treatments, HOLN‐NPs can more effectively enhance the protein expression level of p‐CREB in R28^Glu^ cells. The abovementioned results collectively confirmed that HOLN‐NPs could protect R28^Glu^ cells from death by activating the CaMKII/CREB signaling pathway. Finally, to detect DNA damage in cells, the expression of γ‐H2AX (green fluorescence) was detected in different groups by immunofluorescence staining (Figure 4F,G). Confocal results showed that R28^Glu^ cells showed strong green fluorescence, but the intranuclear green fluorescence was significantly weakened after Nico, OA, and N + O treatment. Notably, the R28^Glu^ cells treated with HOLN‐NPs showed the weakest green fluorescence in their nuclei, ≈5% of the green fluorescence intensity in R28^Glu^ cells. Compared with other treatments, HOLN‐NPs could inhibit the expression of γ‐H2AX more effectively. The above results confirm that HOLN‐NPs protect R28^Glu^ cells from death not only by reactivating the CaMKII/CREB signaling pathway but also by inhibiting the expression of γ‐H2AX.

To verify that HOLN‐NPs protect R28 cells from glutamate damage by activating the CaMKII/CREB pathway, we conducted additional experiments. KN‐93, as a p‐CaMKII inhibitor, has been widely used, and 666‐15 is also a widely recognized p‐CREB inhibitor. The results showed that the protein expression levels of p‐CaMKII and p‐CREB were significantly downregulated after treatment with KN‐93 and 666‐15 in the HOLN‐NPs + R28^Glu^ group (Figure S15, Supporting Information). First, we assessed the cell viability of R28^Glu^ group, HOLN‐NPs + R28^Glu^ group, HOLN‐NPs + R28^Glu^ + KN‐93 group, and HOLN‐NPs + R28^Glu^ + 666‐15 group by CCK‐8 (Figure S16, Supporting Information). The results showed that HOLN‐NPs significantly improved the cell viability of R28^Glu^, consistent with our previous experimental results. After intervention with KN‐93 and 666‐15, the cell viability of the HOLN‐NPs + R28^Glu^ group significantly decreased, indicating that inhibiting p‐CaMKII and p‐CREB reduces the cell viability of R28^Glu^ enhanced by HOLN‐NPs. Second, the live/dead cell assay (AM/PI) also confirmed that after intervention with KN‐93 and 666‐15, the cell death rate of HOLN‐NPs + R28^Glu^ significantly increased (Figure S17, Supporting Information). In summary, the anti‐apoptotic effect of HOLN‐NPs treatment on Glaucoma is mediated by activating CaMKII/CREB signaling pathway.

### 2.5 HOLN‐NPs can Protect Mitochondria and Inhibit the Activation of the Cyt C/PARP Signaling Pathway

**Figure 5:**
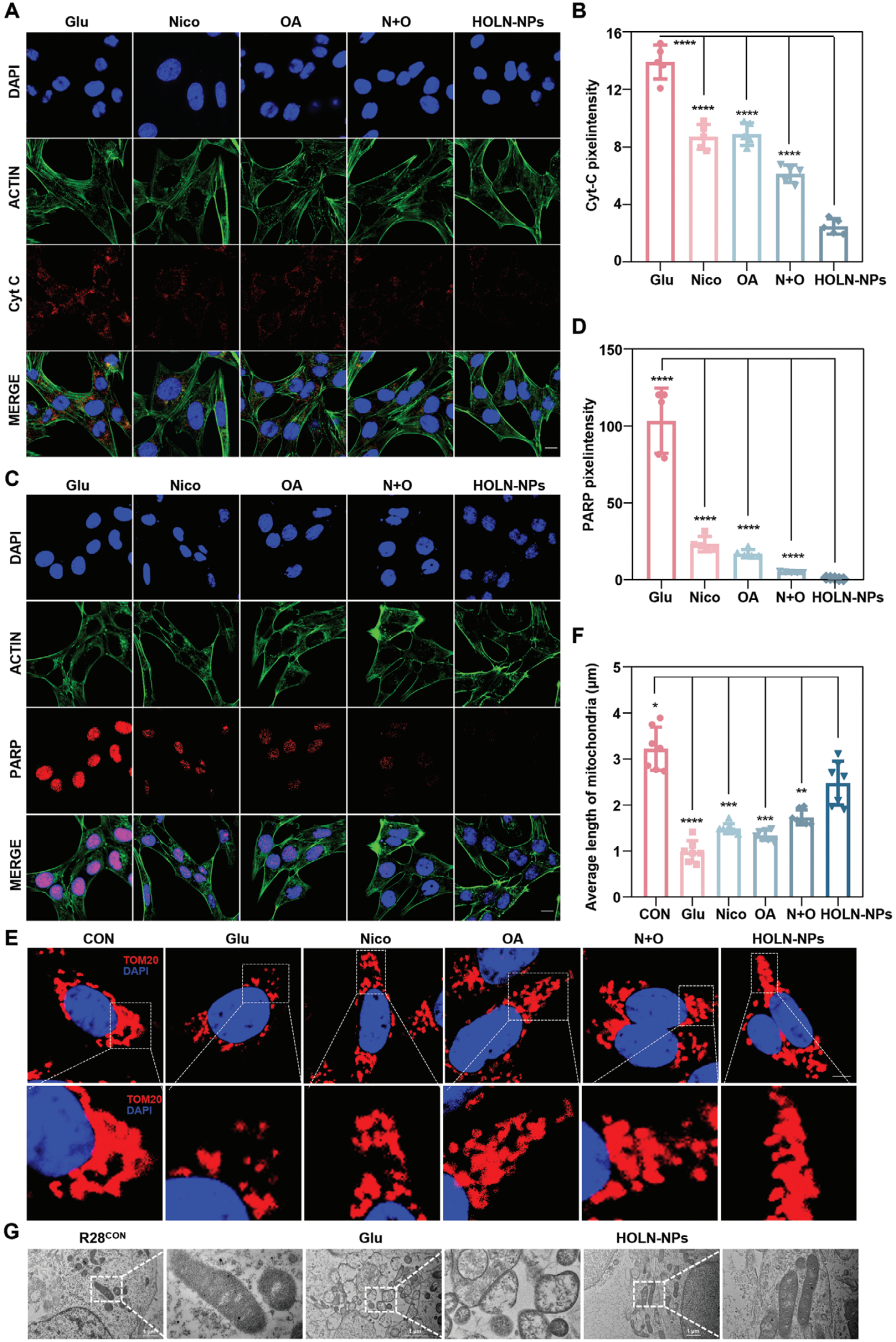
HOLN‐NPs could protect mitochondria and inhibit the activation of the Cyt C/PARP signal pathway. A) The expression of Cyt C in R28^Glu^ with various treatments by IF. Scale bar = 10 µm. B) Relative pixel intensity of Cyt C in R28^Glu^ with various treatments. C) The expression of PARP in R28^Glu^ with various treatments by IF. Scale bar = 10 µm. D) The relative pixel intensity of PARP in R28^Glu^ with various treatments. E) Confocal microscopy was used to determine mitochondrial morphology using the Tom20 antibody (red) via IF. Scale bar = 5 µm. F) The average mitochondrial length in R28^Glu^ with various treatments was analyzed. G) The SEM images of mitochondria in R28^CON^, R28^Glu^, and HOLN‐NPs. Data are presented as the mean ± SD (*n* = 5). Statistical significance was calculated via one‐way ANOVA analysis. *p < 0.05, **p < 0.01, ***p < 0.001, and ****p < 0.0001.

To investigate whether HOLN‐NPs can effectively protect mitochondria in RGCs, Cytochrome C (Cyt C, red fluorescence) expression levels in R28 cells were qualitatively and semi‐quantitatively analyzed by immunofluorescence staining (**Figure** **5**A,B). Cyt C is an essential protein in the electron respiratory chain. During cell apoptosis, Cyt C is released from mitochondria into the cytoplasm and serves as a key regulatory factor for cell apoptosis.^[^ ^27^, ^28^, ^29^ ^]^ Confocal images showed that R28^Glu^ cells showed strong red fluorescence, while R28^Glu^ cells treated by Nico, OA, and N + O showed significantly decreased intracellular red fluorescence. However, HOLN‐NPs‐treated R28^Glu^ cells had the weakest red fluorescence intensity in the cytoplasm, which was approximately one‐seventh of that in R28^Glu^ cells, indicating that HOLN‐NPs could more strongly inhibit the expression of Cyt C protein. Subsequently, the expression of intracellular PARP protein (red fluorescence) was detected by confocal (Figure 5C,D). Nico is a precursor of NAD^+^, which can protect RGC by inhibiting the expression of the PARP.^[^ ^30^, ^31^, ^32^ ^]^ The results showed that R28^Glu^ cells showed strong red fluorescence, and the red fluorescence of R28^Glu^ cells was significantly weakened after Nico, OA, and N + O treatment. HOLN‐NPs treated R28^Glu^ cells showed the weakest intracellular red fluorescence, which was ≈1% of R28^Glu^ cells. Compared with other treatments, HOLN‐NPs can inhibit the protein expression of RARP more effectively. The results above confirm that HOLN‐NPs could significantly reduce the expression levels of Cyt C and apoptosis marker protein PARP, thereby inhibiting R28^Glu^ cell death.

To further investigate whether HOLN‐NPs affect the morphology of mitochondria, immunofluorescence staining was performed on mitochondria in R28 cells by mitochondrial marker Translocase Of Outer Mitochondrial Membrane 20 (TOM20) (red fluorescence) (Figure 5E). The results showed that most of the mitochondria in R28^Glu^ cells after HOLN‐NPs intervention had the typical rod‐shaped and elongated characteristics of normal mitochondria. At the same time, the mitochondria in R28^Glu^ cells without intervention showed small circular or fragmented shapes. The mitochondrial length was analyzed, and the results showed that the mitochondria in R28^Con^ cells was ≈3.2 µm in length, while that in R28^Glu^ cells was ≈0.9 µm (Figure 5F). After treatment with Nico, OA, and N + O, the average length of mitochondria in R28^Glu^ cells increased. In contrast, after treatment with HOLN‐NPs, the average length of mitochondria in R28^Glu^ cells was the longest, ≈2.5 µm. The TEM images of the ultrastructure of mitochondria also confirmed that although the mitochondrial length in R28^Glu^ cells treated with HOLN‐NPs was shorter than R28^CON^, it was significantly longer than that in R28^Glu^ cells. The mitochondrial structure was intact, and the mitochondrial cristae were arranged neatly and clearly in R28^Glu^ treated by HOLN‐NPs (Figure 5G). These results confirm that HOLN‐NPs protected RGCs from death by maintaining mitochondrial morphology and health.

### 2.6 HOLN‐NPs Protect RGC In Vivo

**Figure 6:**
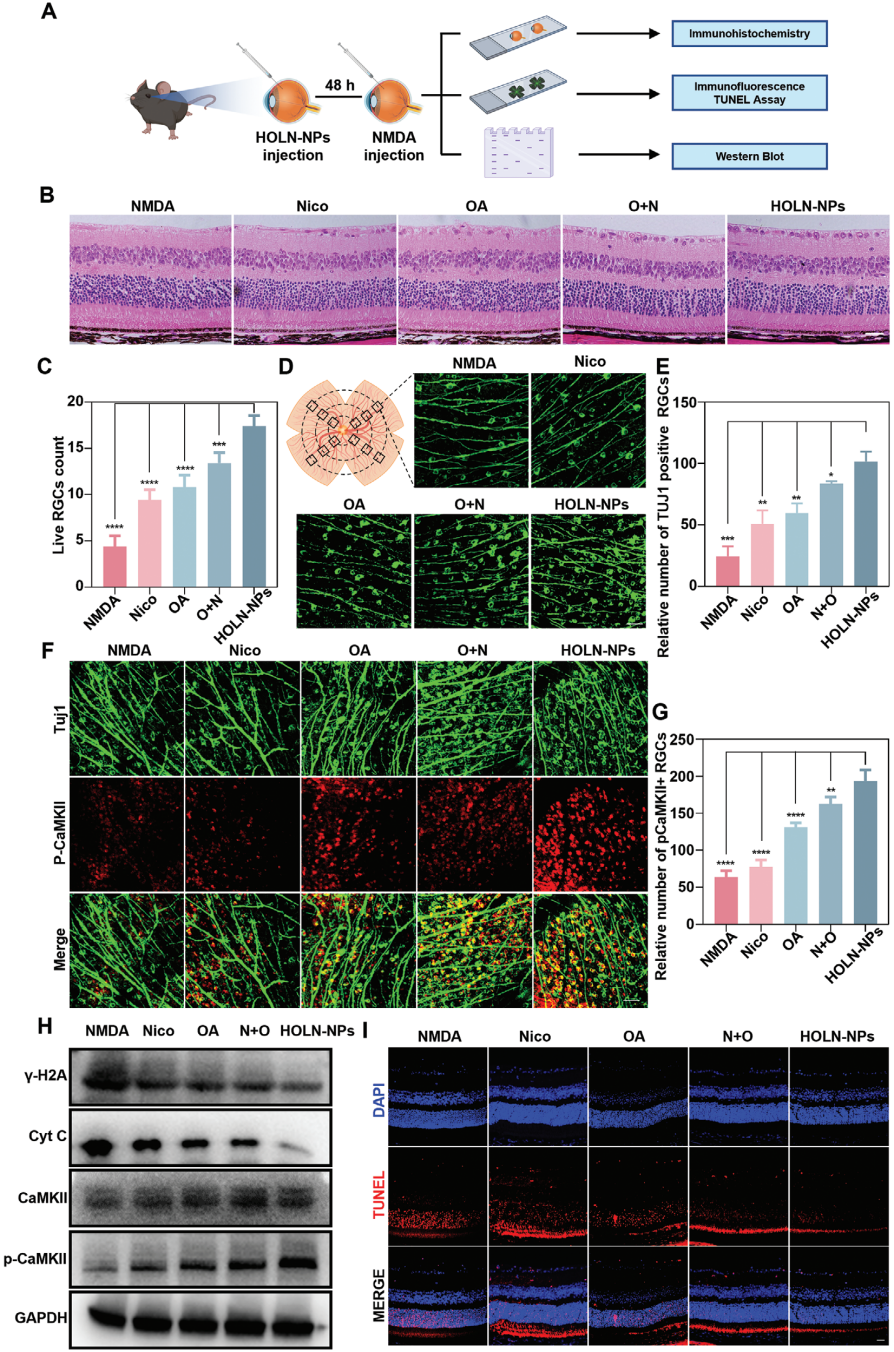
HOLN‐NPs can synergistically inhibit apoptosis of RGC in vivo. A) A mice^NMDA^ model was constructed two days after the intravitreal injection of HOLN‐NPs. On the 14th day after the construction of the model, the mice were sacrificed, and the retina was removed for IF, H&E, and WB. B) H&E staining of the retina of mice^NMDA^ treated with Nico, OA, N + O, and HOLN‐NPs. Scale bar = 50 µm. C) The semi‐quantification of live mice^NMDA^ RGCs after different treatments via H&E staining. D) TUJ‐1 labeled RGCs in flattening of mice^NMDA^ retina treated with Nico, OA, N + O, and HOLN‐NPs. Scale bar = 20 µm. E) The semi‐quantification of TUJ‐1 positive RGCs in flattening of mice retina. F) Confocal images of mice^NMDA^ retinal whole mounts showing p‐CaMKII in TUJ‐1‐labeled RGCs at 12 h after treating with Nico, OA, N + O, and HOLN‐NPs. Scale bar = 20 µm. G) The number of p‐CaMKII^+^/Tuj1^+^RGCs in mice^NMDA^, Nico, OA, N + O, and HOLN‐NPs group. H) The expression level of γ‐H2A, Cyt C, CaMKII, and p‐CaMKII in the retina with different treatments by WB. I) Retinal paraffin sections were stained using TUNEL (red) to observe cell apoptosis. Scale bar = 20 µm. Data are presented as the mean ± SD (*n* = 5). Statistical significance was calculated via one‐way ANOVA analysis. *p < 0.05, **p < 0.01, ***p < 0.001, and ****p < 0.0001.

A glutamate excitotoxicity glaucoma mouse model (mice^NMDA^) was constructed to validate the inhibitory effect of HOLN‐NPs on RGC death in vivo (**Figure** **6**A). First, we injected HOLN‐NPs labeled with fluorescent dye Cy5.5 into the eye through the vitreous cavity to observe their accumulation in retinal RGC. The result showed the strongest red fluorescence signal in RGCs on day 2, significantly stronger than that on day 1 and day 7 (Figure S18, Supporting Information). These results indicated that HOLN‐NPs were rapidly targeted and accumulated in retinal RGCs. Concurrently, we also demonstrated that 500 µm HOLN‐NPs can significantly protect RGCs in vivo by labeling Beta III Tubulin (TUJ‐1) positive RGCs with retinal whole mounts (Figure S19, Supporting Information). Second, we performed immunohistochemical hematoxylin and eosin staining (H&E) analysis on the eyeball tissue sections of each experimental group (Figure 6B,C). The results showed the lowest number of live RGCs at ≈4 per unit scanning area. The number of surviving RGCs in the retina of mice^NMDA^ treated by Nico (≈9), OA (≈10), and N + O (≈13) was significantly more than that of mice^NMDA^. Of note, the number of live RGCs (≈18) after HOLN‐NPs treatment was significantly higher than that in other groups. These results indicate that HOLN‐NPs inhibited the damage of NMDA on retinal RGCs, and their protective effect was substantially stronger than that of Nico, OA, and N + O. Third, retinal RGCs were specifically labeled with TUJ‐1 (green), and the TUJ‐1 positive RGCs on retinal whole mounts in each experimental group were captured by confocal microscopy (Figure 6D,E). The number of TUJ‐1 positive cells per unit imaging area in the mice^NMDA^ retina was the lowest (≈24), while that in the mice^NMDA^ retina after HOLN‐NPs treatment was the highest (≈100) compared with that in the mice^NMDA^ treated with Nico, OA, and N + O. The above results also indicate that HOLN‐NPs were more effective in protecting retinal RGCs from NMDA damage.

Meanwhile, we also evaluated the protective effect of HOLN‐NPs on retinal RGCs in optical nerve crush model mice (mice^ONC^) (Figure S20, Supporting Information). The results showed that the number of TUJ‐1 positive RGCs in the HOLN‐NPs group was the highest compared with that in Nico, OA, and N + O treatment groups, which verified the significant protective effect of HOLN‐NPs on RGCs from multiple animal models. Subsequently, we detected the number of p‐CaMKII (red fluorescence) positive and TUJ‐1 (green fluorescence) positive RGCs in the retina of each group, and the results showed that the number of mice^NMDA^ retinal red and green fluorescence co‐staining RGCs was the least. After Nico, OA, and N + O treatments, the number of mice^NMDA^ retinal RGCs co‐stained by red and green fluorescence increased significantly (Figure 6F,G). The number of RGCs co‐stained with red and green fluorescence in the retina of mice ^NMDA^ treated with HOLN‐NPs was the highest, which was three times higher than that of the untreated mice NMDA group. We also detected the number of retinal p‐CaMKII positive and TUJ‐1 positive RGCs in each group of mice^ONC^ (Figure S21, Supporting Information). The results showed that HOLN‐NPs treated mice^ONC^ had the highest number of red and green fluorescent co‐stained RGCs in the retina among all intervention groups. These results indicated that HOLN‐NPs significantly increased the protein expression level of p‐CaMKII in retinal RGCs. The reactivation effect of HOLN‐NPs was considerably stronger than the other three interventions.

To investigate the regulation of the Cyt C/PARP signaling pathway and CaMKII/CREB signaling pathway‐related proteins in each group, we extracted proteins from retinal tissue for western blot (WB) (Figure 6H). The findings indicate that the expression levels of γ‐H2AX and Cyt C in the retina of mice^NMDA^ were the highest. However, following the administration of Nico, OA, and N + O treatments, the expression levels of these two proteins were significantly lower in the retina of mice^NMDA^. The expression levels of γ‐H2AX and Cyt C in the mice^NMDA^ retina were the lowest after HOLN‐NPs treatment. These results indicate that HOLN‐NPs can effectively protect retinal RGCs by inhibiting the expression of γ‐H2AX and Cyt C. The expression levels of p‐CaMKII and p‐CREB proteins in the mice^NMDA^ retina were the lowest. In contrast, the expression levels of these two proteins were significantly higher in mice^NMDA^ after Nico, OA, and N + O treatment. The protein expression levels of p‐CaMKII and p‐CREB in the retina in mice^NMDA^ treated with HOLN‐NPs were the highest. After treatment with KN‐93 and 666‐15, the protective effect of HOLN‐NPs on RGCs significantly decreased in mice^NMDA^(Figure S22, Supporting Information). These results confirmed that HOLN‐NPs protected retinal RGCs from damage by activating the CaMKII/CREB pathway. Finally, to ensure the protective function of HOLN‐NPs on the retina, retinal sections were stained by TUNEL (Figure 6I). The results showed that the number of red fluorescent staining cells in the outer nuclear layer of the mice^NMDA^ retina was the largest. In contrast, the number of red fluorescent staining cells of the mice^NMDA^ retina after Nico, OA, and N + O treatment was significantly reduced. After treatment with HOLN NPs, the number of red fluorescent staining cells in the outer nuclear layer of the mice^NMDA^ retina was the smallest. At the same time, we also found that HOLN‐NPs exhibited a significant protective effect on the RGCs of mice^IR^ (Figure S23, Supporting Information). The above results confirm that HOLN‐NPs had a significant protective effect on the retina and were not found to have adverse effects on mice eyes (Figure S24, Supporting Information).

### 2.7 HOLN‐NPs Protect RGC Axons from Damage In Vivo

**Figure 7:**
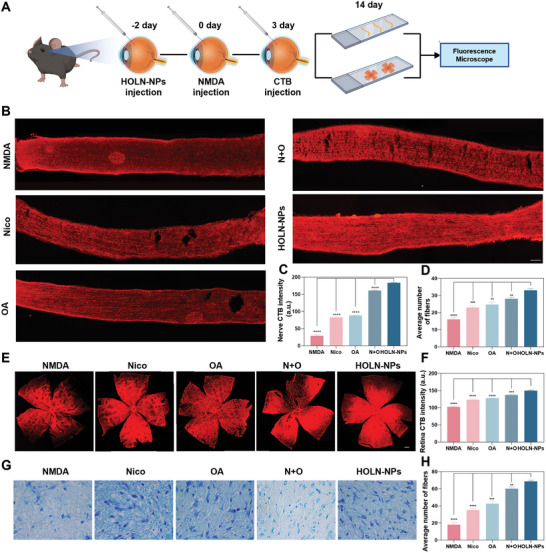
HOLN‐NPs can protect RGC axons from damage in glaucoma mice. A) A mice^NMDA^ model was constructed two days after intravitreal injection of HOLN‐NPs. CTB (Alexa Fluor 555) was injected into the vitreous cavity on the third day after model construction. On the 14th day after the construction of the model, the mice were sacrificed, and their retinas and optic nerves were removed. The red fluorescence intensity of the retina and optic nerve was observed by a fluorescence microscope, and the number of nerve fibers of the optic nerve was calculated. B) Fluorescence images of anterograde CTB tracing of RGC axons in the optic nerve from mice^NMDA^, Nico, OA, N + O, and HOLN‐NPs eyes. Scale bar = 10 µm. C) Quantification of CTB intensity in the optic nerves. D) Quantification of CTB‐positive fibers in the optic nerve. E) Whole‐mount retinal fluorescence images showing CTB filling in the retina. Scale bar = 200 µm. F) Quantification of CTB intensity in the whole‐mount retinas. G) HOLN‐NPs intervention protected from optic nerve damage as assessed by TB staining. H) Quantification of the number of fibers in the optic nerve. Statistical significance was calculated via one‐way ANOVA analysis. *p < 0.05, **p < 0.01, ***p < 0.001, and ****p < 0.0001.

The RGC axon is the only way to transmit visual information from the retina to the brain.^[^ ^33^ ^]^ As RGC axons rarely regenerate after injury, the degeneration of RGC axons leads to permanent vision loss.^[^ ^34^ ^]^ Preserving the integrity of the RGC axon is essential for vision protection. To investigate whether HOLN‐NPs can protect RGC axons, we injected Alexa Fluor 555 conjugated Cholera Toxin subunit B (CTB) into the vitreous of mice to track the RGC axons anterograde (**Figure** **7**A). One week after the construction of the mice^NMDA^ model, RGC axons were severely damaged, the fluorescence intensity of CTB labeling of the optic nerve was ≈20.50 a.u., and the number of nerve fibers was ≈16 a.u. After Nico, OA, and N + O treatment, the fluorescence intensity of CTB‐labeled optic nerve was ≈82.68, ≈88.40, and ≈161.01, and the number of nerve fibers was ≈23, ≈25, and ≈28, respectively. In contrast, most axons were protected from NMDA toxicity after HOLN‐NPs treatment, while the fluorescence intensity of CTB‐labeled axons was ≈183.37, and the number of nerve fibers was ≈33 (Figure 7B–D). These results indicated that HOLN‐NPs effectively protected the axons of RGC from NMDA damage.

To verify the above results, fluorescence microscopy was performed on the retinas of mice in each group injected with CTB. The fluorescence intensity of CTB‐Alexa Fluor 555 labeled retina was analyzed to evaluate the axon survival of RGCs in each experimental group. The results showed that the fluorescence intensity of the retina in the mice^NMDA^ group was ≈102.48. After Nico, OA, and N + O treatment, the fluorescence intensity of mice^NMDA^ retina was ≈123.64, ≈128.29, and ≈137.33, respectively. The fluorescence intensity of mice^NMDA^ retina was the strongest after HOLN‐NPs treatment (≈150.12) (Figure 7E,F).

To confirm the protective effect of HOLN‐NPs on the optic nerve, Toluidine Blue (TB) staining was performed after longitudinal resection of the optic nerve, to calculate the number of nerve fibers and the degree of damage in each group. TB can stain the myelin sheath of nerves, and this staining method is mainly used to show the morphological structure and pathological changes of the myelin sheath of nerves. The results showed that the RGC axons of mice^NMDA^ suffered the most severe damage, while the RGC axonal damage was reduced after Nico, OA, and N + O treatments. HOLN‐NPs effectively reduced RGC axon damage, and the number of axons was ≈3.8 times that of mice^NMDA^ (Figure 7G,H). We have evaluated the fluorescent intensity of CTB‐labeled neurons in the lateral geniculate nucleus (LGN) or Superior colliculus (SC) (Figure S25, Supporting Information). The results showed that the red fluorescence intensity of LGN and SC was significantly reduced after NMDA treatment. However, after intervention with HOLN‐NPs, the red fluorescence intensity of LGN and SC was significantly enhanced (Figure S25, Supporting Information). In summary, our results suggest that HOLN‐NPs can not only effectively protect the cell body of RGCs, but also strongly maintain the integrity of the distal projection of the RGC axons, which is necessary for the ultimate maintenance of visual function.

### 2.8 HOLN‐NPs Protect the Visual Function of Glaucoma Mice

**Figure 8:**
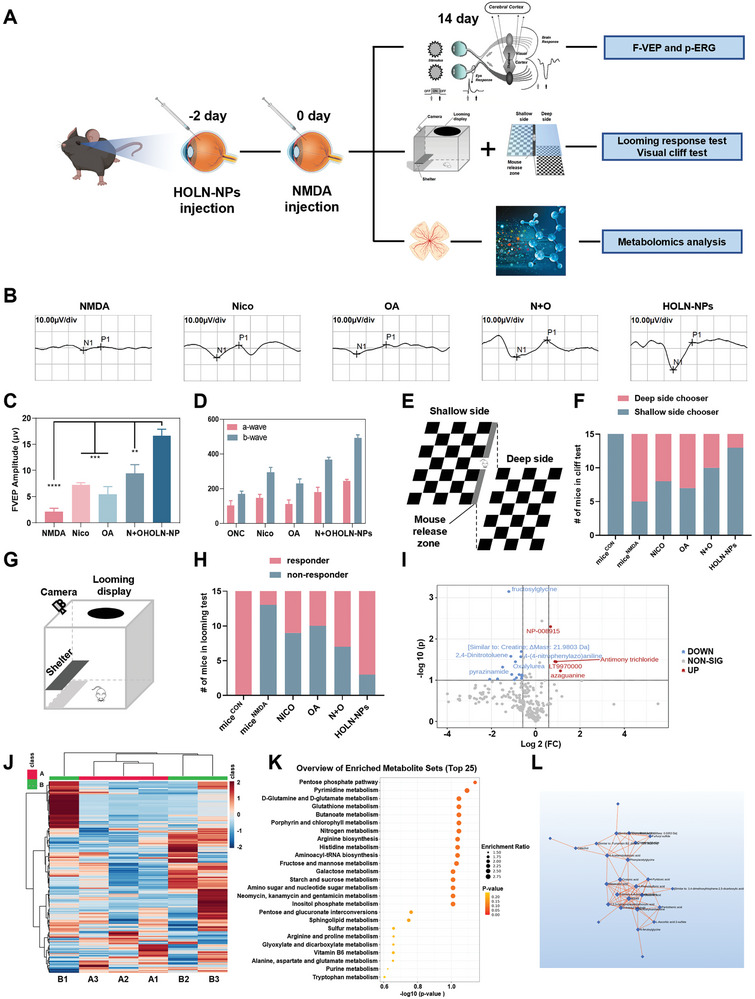
Visual function and metabolomics analysis of HOLN‐NPs in *vivo*. A) HOLN‐NPs were injected into the vitreous cavity 2 days before the mice^NMDA^ model was constructed. The visual function of mice was tested 14 days after the model was constructed. Metabolomic analysis was performed on the retinas of mice in each group. B) The FVEP results of mice after different treatments. C) The quantification of F‐VEP amplitude of mice. D) The quantification of N1 and P1 wave amplitude of mice. E) Schematic diagram of the visual cliff test. F) Visual cliff performance, from mice^NMDA^, Nico, OA, N + O, and HOLN‐NPs treated mice. Data showed the number of shallow/deep side choosers. G) Schematic diagram of the looming response test. H) Performance in response to looming stimuli, from mice^NMDA^, Nico, OA, N + O, and HOLN‐NPs treated mice. Data showed the number of responders and non‐responders. I) Volcanic maps showed differences in retinal metabolites between the mice^NMDA^ and the HOLN‐NPs group. J) A heat map showed unsupervised hierarchical clustering of metabolites quantified by LC‐MS. K) The dot plot depicted the top‐25 KEGG pathways in retinas between the mice^NMDA^ and the HOLN‐NPs group. The size of the dots corresponds to the enrichment ratio, and the color corresponds to the correlated p‐value. L) The interaction of metabolites in retinas between the mice^NMDA^ and the HOLN‐NPs group. Data are presented as the mean ± SD (*n* = 3). Statistical significance was calculated via one‐way ANOVA analysis. **p < 0.01, ***p < 0.001, and ****p < 0.0001.

After leaving the eye, visual information travels through several relay centers of the brain such as LGN and SC, and ultimately reaches the primary visual cortex.^[^ ^35^ ^]^ We next tested whether preserved RGC responses could be transmitted to the primary visual cortex in vivo. Subsequently, we evaluated the protective effect of HOLN‐NPs on visual function in glaucoma model mice by Flash‐Visual Evoked Potential (F‐VEP) (**Figure** **8**A). Results showed that the P‐wave amplitude of the F‐VEP of mice^NMDA^ was ≈2.16 µV, which was significantly lower than that of mice^NMDA^ treated with Nico(≈7.25 µV), OA(≈5.46 µV), and N + O (≈9.46 µV). However, after HOLN‐NPs treatment, the P‐wave amplitude of the F‐VEP in mice^NMDA^ was ≈16.63 µV, which was significantly higher than that in the other experimental groups (Figure 8B,C). The results of the F‐VEP examination thus indicated that HOLN‐NPs could more effectively protect the conduction function from RGCs to the visual center.

To further evaluate the protective effect of HOLN‐NPs on visual function, Flash‐Electroretinogram (F‐ERG) was used to detect retinal RGC function in each experimental group in the mice^ONC^ animal model (Figure 8D; Figure S26, Supporting Information). Two weeks after the mice^ONC^ model was constructed, the a‐wave amplitude of F‐ERG was ≈103.91 µV and the b‐wave amplitude was ≈170.74 µV, which indicated that the optic nerve injury could lead to the serious loss of RGC function. After the mice^ONC^ was treated with Nico, OA, and N + O, the a‐wave amplitude (≈147.18, ≈112.97, and ≈179.92 µV) and b‐wave amplitude (≈294.40, ≈229.52, and ≈367.27 µV) of F‐ERG increased. Notably, the a‐wave amplitude (≈245.90 µV) and b‐wave amplitude (≈492.34 µV) of F‐ERG in mice^ONC^ after HOLN‐NPs treatment were 2.4 times that of mice^ONC^, and b‐wave amplitude were 2.9 times that of mice^ONC^. Both a‐wave and b‐wave amplitude improved significantly.

We carried out vision‐based behavioral tests to test whether HOLN‐NPs‐mediated protection of the visual pathway indeed preserves vision. First, we performed the visual cliff test to assess the maintained ability to discriminate visual depth after HOLN‐NPs treatment.^[^ ^17^ ^]^ This test is based on a mouse's innate tendency to avoid the deep side and step onto the shallow side of a visual cliff. Mice were placed on the center platform between the deep and shallow sides of the cliff, and their choices to step toward either side were recorded (Figure 8E,F). In the mice^Con^ group, all 15 mice chose the shallow side. Significantly worse performance was recorded in mice^NMDA^, with 5 out of 15 mice choosing the shallow side. After Nico, OA, and N + O treatment, 8 out of 15 mice^NMDA^, 7 out of 15 mice^NMDA^, and 10 out of 15 mice^NMDA^ chose the shallow side. By contrast, 13 out of 15 mice mice^NMDA^ treated with HOLN‐NPs chose the shallow side.

Next, we evaluated the innate defensive responses of mice to looming visual stimuli representing environmental threats.^[^ ^17^ ^]^ The looming experiment was conducted in an enclosure with an overhead monitor to display looming stimuli, a shelter for the mouse to hide, and a camera to record the behavior of the mouse. The following behaviors: freezing, fleeing to the shelter, and tail rattling, were displayed consistently in mice with normal vision in response to looming stimuli. Consequently, we recorded the mouse as a responder to looming stimuli if it reacted with one or more of these behaviors (Figure 8G,H). In the mice^Con^ group, all the 15 mice were responders. In the mice^NMDA^ group, only 2 out of 15 mice responded to looming stimuli. Notably, 12 out of the 15 HOLN‐NPs‐treated mice^NMDA^ were responders, and the number of responders is six times that of the mice^NMDA^ group. Taken together, our results demonstrate for the first time that HOLN‐NPs protect RGCs and preserve visual function through mitochondrial protection and reactivation of the p‐CaMKII/p‐CREB pathway in vivo.

### 2.9 Effects of HOLN‐NPs on Retinal Metabolism in Glaucoma Mice

To further investigate the metabolic regulation mechanism of HOLN‐NPs on the retina of mice^NMDA^, metabolomics analysis of metabolites in mice^NMDA^ and HOLN‐NPs‐treated mice^NMDA^ retina was performed by liquid chromatography/mass spectrometry (LC‐MS). First, we performed principal component analysis (PCA) on 4063 quantitative metabolites isolated from the retina. The results showed that principal components 1 (PC1) and 2 (PC2) accounted for 52.7% and 26.8% of all detectable metabolites, respectively (Figure S27, Supporting Information). Second, the Pearson correlation analysis was conducted for metabolites in the two groups, and the results showed a close correlation between samples (Figure S28, Supporting Information). Subsequently, we represented the differences in metabolites between different groups through heat maps, which revealed the metabolic reprogramming of the retina of mice^NMDA^ by HOLN‐NPs (Figure 8I). The results showed that compared with those in the HOLN‐NPs group, the levels of fructosylglycine, Creatine analog, 2,4‐Dinitrotoluene, 4‐(4‐nitrophenylazo) aniline, Oxalylurea, Pyrazinamide, etc. in the retina of mice^NMDA^ were significantly reduced, while the levels of NP‐008915, Antimony trichloride, LT9970000, Azaguanine were increased considerably. Next, KEGG enrichment analysis of retinal metabolites was performed in mice^NMDA^ and HOLN‐NPs treated mice^NMDA^. The results demonstrated that the Pentose phosphate pathway, Pyrimidine metabolism, D‐Glutamine, and D‐glutamate metabolism, Metabolites related to Glutathione metabolism, and Butanoate metabolism pathways were significantly enriched in retinas of the HOLN‐NPs group (Figure 8J,K). We also analyzed the interaction between different metabolites (Figure 8L; Figure S29, Supporting Information). Overall, HOLN‐NPs protected RGCs by metabolically reprogramming mice^NMDA^ retinas via the regulation of the Pentose phase pathway, Pyrimidine metabolism, D‐Glutamine and D‐glutamine metabolism, Glutathione metabolism, and Butanoate metabolism pathway.

## 3 Conclusion

ROS clearance, mitochondrial protection, and CaMKII/CREB pathway activation are promising treatments for glaucoma.^[^ ^36^, ^37^, ^38^, ^39^ ^]^ In this study, we developed hypoxia and ROS dual response nano drugs (HOLN‐NPs), which can release Nico and OA under hypoxic environments and ROS conditions. In vitro studies demonstrated HOLN‐NPs scavenged ≈70% of H_2_O_2_ and ≈90% of ∙ABTS^+^, showing excellent ROS scavenging ability. Moreover, HOLN‐NPs inhibited R28^Glu^ cell apoptosis through mitochondrial protection and activation of the CaMKII/CREB pathway. In vivo studies further revealed that HOLN‐NPs efficiently scavenged ROS from the retina of mice^NMDA^, while simultaneously protecting mitochondria and reactivating the CaMKII/CREB pathway to protect RGCs from death. The protection rate was approximately twice as high as nicotinamide and 1.7 times as high as oleic acid. In addition, visual function and behavioral experiments demonstrated that HOLN‐NPs exerted superior protection on the visual function of glaucoma model mice. This effect was attributed to the metabolic reprogramming of the retina through regulating the Pentose phase pathway, Pyrimidine/D‐Glutamine/Glutathione/Butanoate metabolism pathway. Well, there are studies indicating that ROS has relations with neurodegenerative diseases, such as Alzheimer's, Parkinson's, Huntington, and Amyotrophic lateral sclerosis. Overall, the findings of this study not only propose HOLN‐NPs as a novel strategy for optic nerve protection in glaucoma but also indicate potential applicability for the treatment of other neurodegenerative diseases.

## Conflict of Interest

The authors declare no conflict of interest.

## Supporting information

Supporting Information

## Data Availability

The data that support the findings of this study are available on request from the corresponding author. The data are not publicly available due to privacy or ethical restrictions.
