# Supplementary material for: Self‐Assembly Hypoxic and ROS Dual Response Nano Prodrug as a New Therapeutic Approach for Glaucoma Treatments
Source: Adv Sci (Weinh). 2024 Sep 4;11(41):2407043. doi: 10.1002/advs.202407043 (PMC11538650; doi:10.1002/advs.202407043)
Supplement: Supplementary file 1 — Supporting Information [file ADVS-11-2407043-s001.docx]

**Supporting information**

**Self-assembly hypoxic and ROS dual response nano prodrug as a new therapeutic approach for glaucoma treatments**

*Xuezhi Zhou*, *Rong Rong*, *Ganghao Liang*, *Yukun Wu,* *Chun Xu*, *Haihua Xiao*, *Dan Ji**, *Xiaobo Xia**

Dr. X. Zhou, Dr. R. Rong, Dr. Y. Wu, Prof. D. Ji, Prof. X. Xia

Central South University, Changsha, Hunan, 410008, P.R. China

Hunan Key Laboratory of Ophthalmology, Changsha, Hunan, 410008, P.R. China

National Clinical Research Center for Geriatric Diseases, Central South University, Changsha, Hunan, 410008, P.R. China

E-mails: [gree1333@csu.edu.cn](mailto:gree1333@csu.edu.cn), [xbxia21@csu.edu.cn](mailto:xbxia21@csu.edu.cn)

G. Liang, Prof. H. Xiao

Beijing National Laboratory for Molecular Sciences, State Key Laboratory of Polymer Physics, Institute of Chemistry, Chinese Academy of Sciences, Beijing, 100190, P. R. China.

University of Chinese Academy of Sciences, Beijing 100049, P. R. China

E-mails: [hhxiao@iccas.ac.cn](mailto:hhxiao@iccas.ac.cn)

Prof. C. Xu

School of Dentistry, The University of Queensland, Brisbane, 4006, Australia

**TABLE OF CONTENTS**

**MATERIALS AND METHODS…………………………………………………………….….2-12**

**SUPPLEMENTAL FIGURES, SCHEMES, AND TABLES**

Figure S1………………………………………………………………………………………........13

Figure S2……………………………………………………………………………………............14

Figure S3…………………………………………………………………………………..…..........15

Figure S4………………………………………………………………………………………........16

Figure S5…………………………………………………………………………………..…..........17

Figure S6…………………………………………………………………………………..…..........18

Figure S7…………………………………………………………………………………..……......19

Figure S8…………………………………………………………………………………..…..........20

Figure S9…………………………………………………………………………………............... 21

Figure S10………………………………………………………………………………….....….....22

Figure S11…………………………………………………………………………………..…........23

Figure S12…………………………………………………………………………………..…........24

Figure S13…………………………………………………………………………………..…........25

Figure S14…………………………………………………………………………………..….…...26

Figure S15…………………………………………………………………………………..….…...27

Figure S16…………………………………………………………………………………..….…...28

Figure S17…………………………………………………………………………………..….…...29

Figure S18…………………………………………………………………………………..….…...30

Figure S19…………………………………………………………………………………..….…...31

Figure S20…………………………………………………………………………………..….…...32

Figure S21…………………………………………………………………………………..….…...33

Figure S22…………………………………………………………………………………..….…...34

Figure S23…………………………………………………………………………………..….…...35

Figure S24…………………………………………………………………………………..….…...36

Figure S25…………………………………………………………………………………..….…...37

Figure S26…………………………………………………………………………………..….…...38

Figure S27…………………………………………………………………………………..….…...39

Figure S28…………………………………………………………………………………..……....40

References…………………………………………………………………………………..….…...41

**EXPERIMENTAL SECTION**

**Materials and equipment**

4-dimethylaminopyridine (DMAP), anhydrous dichloromethane, N-(3-dimethylaminopropyl)-N'-ethylcarbodiimide hydrochloride (EDCI), oleic acid, nicotinamide, oxalyl chloride, 4-[(E)-2-(4-hydroxyphenyl)diazen-1-yl]phenol, (2-bromoethoxy)-tert-butyldimethylsilane, potassium carbonate (K2CO3), tetrabutylammonium fluoride hydrate (TBAF), methoxy (polyethylene glycol)-5000 (mPEG5k-OH), 1,2,4,5-cyclohexanetetracarboxylic acid dianhydride (CHTA), and anhydrous N,N-dimethylformamide were purchased from Energy Chemical, China. Dulbecco’s modified Eagle’s medium (DMEM) was purchased from Hyclone (UT, USA); Trypsin and serum were purchased from Gibco (Carlsbad, CA). Nicotinamide and Oleic acid were purchased from MedChemExpress (NJ, USA). CCK8 and ROS kits were purchased from Beyotime (Shanghai, China). The hydrogen peroxide test kit was purchased from Beyotime (Shanghai, China). Cell viability (live and dead cell staining) assay kits were purchased from KeyGEN Biotech (KGAF001, Jiangsu, China). Annexin V-FITC Apoptosis Detection Kit I (#556547, BD Pharmingen, USA) was used to assess apoptosis according to the manufacturer’s instructions. Actin-Tracker Green-488 (C2201S, 1:200) was purchased from Beyotime (Shanghai, China). Hypoxyprobe^TM^ Kit was purchased from NPI (Burlington, USA). ROS Assay Kit was purchased from Beyotime (Shanghai, China). The mitochondrial membrane potential assay kit with JC-1 was purchased from Beyotime (Shanghai, China). CaMKII antibody (AF1639, WB: 1:1000) and CREB antibody (AF1018, WB:1:1000) were purchased from Beyotime (Shanghai, China). Brn 3a antibaody (ab245230, IF: 1:100), p-CaMKII antibody (ab171095, IF: 1:500, WB: 1:1000), p-CREB antibody (ab220798, IF: 1:500, WB: 1:1000), γ-H2A.X antibody (ab26350, IF: 1:500, WB: 1:1000), Cyt C antibody (ab133504, IF: 1:500, WB: 1:1000), TOM20 antibody (ab186735, IF: 1:500), and TUJ-1 antibody (ab18207, IF: 1:500) were purchase from Abcam (Cambridge, UK). PARP (AF1657, IF: 1:200) antibody was purchased from Beyotime (Shanghai, China). GAPDH antibody (#2118, WB: 1:1000) was purchased from Cell Signaling Technology (Beverly, MA, USA). Goat anti-mouse Alexa Fluor® Plus 555 (ab150114, 1:500), Goat anti-rabbit Alexa Fluor® Plus 555 (ab150078, 1:500), goat anti-rabbit Alexa Fluor® Plus 488 (ab150077, 1:500), [Goat Anti-Rabbit IgG H&L](https://www.abcam.cn/goat-rabbit-igg-hl-hrp-ab6721.html) (ab6721, 1:2000), and [Goat Anti-Mouse IgG H&L](https://www.abcam.cn/goat-mouse-igg-hl-hrp-ab6789.html) (ab6789, 1:2000) were purchased from Abcam (Cambridge, UK). Western blotting related reagents (RIPA protein lysis buffer, 5× protein loading buffer were purchased from Solarbio. A cocktail of protein phosphatase and protease inhibitors were purchased from Sigma-Aldrich (St Louis, MO).666-15 (CAS No.:1433286-70-7) and KN-93 (CAS No.:139298-40-1) were purchased from MedChemExpress company. ^1^H NMR spectra were run on a 300 or 400 MHz NMR spectrometer (Bruker). ^13^C NMR spectra were measured by a 75 or 101 MHz NMR spectrometer (Bruker). The Size and zeta potential of nanoparticles were measured by a Malvern Zetasizer (Nano ZS, U.K.). High performance liquid chromatography (HPLC) analysis was performed on an Agilent 1200 series instrument. A transmission electron microscope (TEM, Hitachi HT7700, Japan) was used to observe the cells and the morphology of the nanoparticles. The UV absorbance spectra of nanoparticles were measured using a UV-VIS-NIR spectrophotometer (UV-2600). Flow cytometric analysis was performed using a flow cytometer (BD Biosciences, USA) and fluorescence imaging analysis was performed using a confocal laser scanning microscopy (CLSM) (ZEISS LSM 880, Germany). The HE imaging analysis was performed using an inverted fluorescence microscope (Olympus IX 83, Japan). Tanon 5200 Multi fully automated chemiluminescence/fluorescence image analysis system (Shanghai, China) was used for the western blot analysis. Quantitative determination of CCK8 was carried out using a Bio-Rad microplate reader (SpetraMax M3). Electroretinogram (ERG) and visual evoked potential (VEP) detection were performed using the Roland ophthalmic electrophysiological diagnostic system (Germany).

**Methods**

**Synthesis of compound 1**

Oleic acid (4.17 mmol, 1.18 g), DMAP (4.17 mmol, 0.5 g) and EDCI (6.26 mmol, 1.2 g) were dissolved in 9 mL DCM and stirred for 30 min. Then 2,2'-(propane-2,2-diylbis(sulfanediyl))bis(ethan-1-ol) (DSB, 4.59 mmol, 0.9 g) was added to the reaction system and reacted for 2 days. After the reaction was completed, the solvent was removed by a rotary evaporator. The resultant crude product was purified by silica column to afford compound **1**. ^1^H NMR (400 MHz, DMSO-*d*_6_) δ 5.32 (t, *J* = 4.7 Hz, 2H), 4.80 (t, *J* = 5.2 Hz, 1H), 4.15 (t, *J* = 6.8 Hz, 2H), 3.52 (q, *J* = 6.8 Hz, 2H), 2.81 (t, *J* = 6.8 Hz, 2H), 2.66 (t, *J* = 7.0 Hz, 2H), 2.28 (t, *J* = 7.3 Hz, 2H), 1.98 (q, *J* = 6.6 Hz, 4H), 1.54 (s, 8H), 1.32 – 1.22 (m, 20H), 0.90 – 0.81 (m, 3H).

**Synthesis of compound 2 (OLN monomer)**

Nicotinamide (1 mmol, 122 mg) was added to 8 mL DCM. The solution was cooled to 0 ℃ and protected by N_2_. Oxalyl chloride was added dropwise to the reaction system The reaction was then refluxed for 6 h and cooled to room temperature. Compound **1** (1 mmol, 122 mg) was dissolved in 8 mL DCM, added to the dropwise to the above reaction system and reacted for 2 days. After the reaction was completed, the solvent was removed by a rotary evaporator. The resultant crude product was purified by a silica column to afford compound **2** (OLN monomer). ^1^H NMR (300 MHz, DMSO-*d*_6_) δ 11.25 (s, 1H), 8.98 (s, 1H), 8.75 (d, *J* = 4.5 Hz, 1H), 8.20 (d, *J* = 7.9 Hz, 1H), 7.52 (dd, *J* = 7.6, 4.8 Hz, 1H), 5.31 (t, *J* = 4.6 Hz, 2H), 4.29 (t, *J* = 6.8 Hz, 2H), 4.16 (t, *J* = 6.6 Hz, 2H), 2.87 (dt, *J* = 17.6, 6.7 Hz, 4H), 2.26 (t, *J* = 7.3 Hz, 2H), 2.07 – 1.79 (m, 4H), 1.62 – 1.44 (m, 8H), 1.23 (s, 20H), 0.83 (d, *J* = 6.8 Hz, 3H).

**Synthesis of compound 3**

4-[(E)-2-(4-hydroxyphenyl)diazen-1-yl]phenol (0.5 mmol, 107 mg), K_2_CO_3_ (1.5 mmol, 207 mg), and (2-bromoethoxy)-tert-butyldimethylsilane (1.5 mmol, 359 mg) were added to 5 mL acetonitrile. The solution was refluxed for 24 h in an N_2_ atmosphere. After the reaction was completed, the solvent was removed by a rotary evaporator. The resultant crude product was purified by silica column to afford compound **3**. ^1^H NMR (400 MHz, DMSO-*d*_6_) δ 7.83 (d, *J* = 8.9 Hz, 4H), 7.11 (d, *J* = 9.0 Hz, 4H), 4.14 (t, *J* = 4.6 Hz, 4H), 3.95 (t, *J* = 4.6 Hz, 4H), 0.88 (s, 18H), 0.08 (s, 12H).

**Synthesis of compound 4**

Compound 3 (0.29 mmol, 155 mg) was dissolved in 4 mL THF. Then 1M TBAF (1.46 mL) was added to the above solution and stirred for 6 h. After the reaction was completed, the solvent was removed by a rotary evaporator. The resultant crude product was purified by silica column to afford compound **4**. ^1^H NMR (400 MHz, DMSO-*d*_6_) δ 7.83 (d, *J* = 8.9 Hz, 4H), 7.11 (d, *J* = 8.8 Hz, 4H), 4.94 (t, *J* = 5.5 Hz, 2H), 4.08 (t, *J* = 4.9 Hz, 4H), 3.75 (q, *J* = 5.1 Hz, 4H).

**Synthesis of P1**

Compound 4 (0.140 mmol, 42.2 mg) and CHTA (0.147 mmol, 32.8 mg) were dissolved in 5 mL anhydrous DMF. After magnetic stirring at 50 ℃ for 24 h, mPEG_5k_-OH (75 mg) was added to the reaction system and reacted for another 24 h. Subsequently, the mixture was placed in a dialysis bag (MWCO: 8000 Da) and dialyzed against deionized water for 48 h. Subsequently, the solution was freeze-dried under reduced pressure to obtain P1 which was analyzed by ^1^H NMR.

**Nanoparticles Formulation of HOLN-NPs**

Briefly, a solution of compound **2** (1 mg) and **P1** (1 mg) in DMSO (1 mL) was added to deionized water (10 mL) dropwise under vigorous stirring. The mixture was dialyzed in a dialysis bag (MWCO: 8000 Da) for 12 h.

**Antioxidant Activity.** The H_2_O_2_ scavenging activity of HOLN-NPs was evaluated with Hydrogen Peroxide Detection Kit (Beyotime, Shanghai, China). First, HOLN-NPs (500 µM) was incubated in 2 ml of PBS containing 50 mM H_2_O_2_ at room temperature for 0.5 h, 1 h, 4 h, 6 h, 24 h, or various concentrations of HOLN-NPs (from 50 μM, 100 μM, 250 μM to 500 μM) were incubated in 2 ml of PBS containing 50 mM H_2_O_2_ at room temperature for 4 h. Then added 50 µL of samples or standard solution (1, 2, 5, 10, 20, 50 and 100 µM) to a 96-well plate. Next, 100 µL of hydrogen peroxide detection reagent was added to each well. After incubation at room temperature for 30 min, the concentration of remaining H_2_O_2_ was determined by measuring the absorption at 560 nm with a Bio-Rad multiple plate reader (SpetraMax M3), and the H_2_O_2-_ scavenging capacity was calculated. The specific calculation method refers to the previous study.

For the •ABTS^+^ assay, HOLN-NPs (100 μL, 100 μM) were mixed with the •ABTS^+^ solution(100 μL，7 mM) for 0.5 h, 1 h, 3 h, 6 h and 24 h in the dark. Subsequently, the absorbance of the mixture at 734 nm was measured with a Bio-Rad multiple plate reader (SpetraMax M3). The •ABTS^+^ scavenging activity of the HOLN-NPs was calculated based on the following equation

•ABTS^+^ scavenging activity (%) =(A_0_-A_i_)/A_0_×100%

Where A_0_ and A_i_ represented the absorbance of •ABTS^+^ solution before and after adding HOLN-NPs samples.

**Cell culture.** R28 cells, a retinal precursor cell line with the potential for differentiation, can express the specific antigen Thy 1.1 of RGCs and are commonly used to study neuronal function and neuroprotection *in vitro*. R28 cells were cultured in DMEM low-glucose medium supplemented with 10% fetal bovine serum and 1% penicillin-streptomycin solution. The glutamate cytotoxicity model (R28^Glu^) was established as described previously. Briefly, the concentration of glutamic acid was 10 μM and the treatment time was 24 h. Drug intervention was performed 7 h before modeling. R28 cells were cultured in DMEM low-glucose medium supplemented with 10% fetal bovine serum and 1% penicillin-streptomycin solution. The oxygen and glucose deprivation model (R28^OGD^) were established as described previously. Briefly, the culture medium was replaced with serum-free and sugar-free medium and placed in a hypoxic incubator for 4 h. Thereafter, the culture medium was changed with normal medium and cultured under regular conditions until the experiment was performed. Drug intervention was performed 7 h before modeling.

**Transmission electron microscopy.** The nanoparticle sample was prepared by dropping the sample onto a copper mesh and imaged directly after drying. The TEM images were obtained by transmission electron microscopy (HT7700).

**Immunofluorescence**. A cover slide was placed on the bottom of each well in a 24 well plate. R28 cells were seeded in each well overnight at a density of 1×10^5^ cells/well. Then, the cells were treated with Nico (10 μM), OA (1 μM), N+O (10 μM), and HOLN-NPs (500 μM) for 7 h. After R28^Glu^ modeling for 12 h, the R28 cells from different groups were fixed in 4% paraformaldehyde for 15 min. Cells were then washed once with phosphate-buffered saline (PBS) and permeabilized with 0.1% Trixton-X-100 in PBS for 10 min. After blocking with 5% bull serum albumin (BSA) for 30 min, R28 cells were immunostained with the p-CaMKII primary antibody (1:500), p-CREB primary antibody (1:500), γ-H2AX primary antibody (1:500), Cyt C primary antibody (1:500), PARP primary antibody (1:500), and TOM20 primary antibody (1:500) with 5% BSA at 4 ℃ overnight. After washing with PBS three more times, the cells were incubated with the Goat anti-rabbit Alexa Fluor® Plus 555 (1:500) and SF488-labeled phalloidin (1:500) for 60 min. Cells were then washed five times with PBS and stained with 4’,6-diamidino-2-phenylindole (DAPI; Solarbio, Beijing, China). Afterwards, images were taken using CLSM (ZEISS LSM 880, Germany).

Frozen sections of retinal tissues were evaluated for TUJ-1 and p-CaMKII using immunofluorescence. Frozen retinal sections were first warmed to room temperature and fixed with 4% paraformaldehyde. After blocking with 5% BSA for 1 h, the sections were immunostained with TUJ-1 (1:500) and p-CaMKII (1:500) at 4 °C overnight. After washing with PBS three times, the sections were then incubated with anti-rabbit IgG Alexa Fluor 555 (1:500) and goat anti-rabbit Alexa Fluor® Plus 488 (1:500) for 1 h at room temperature and then counterstained with DAPI. After mounting the slides, the results were obtained using an immunofluorescence slice scan (3D HISTECH, Budapest, Hungary).

For immunohistochemistry of retinal whole mounts, the mouse eyeballs were enucleated immediately and placed in an ice-cold PBS solution, and the retinas were carefully dissected and washed. Retinas were then immersed in 4% paraformaldehyde (pH 7.4) for 30 min before being transferred to fresh PBS twice for 5 min. The retina was subsequently incubated in 5% BSA + 0.5% Triton-X-100 for 2 h at room temperature. It was then incubated with TUJ-1 antibody (1:500) overnight at 4 ℃. After washing with PBS by 3 times, the retina was incubated with the goat anti-rabbit Alexa Fluor® Plus 488 (1:500). From this step onwards, all the operations were performed in the dark. The retina was carefully placed on the slides with one drop of anti-fade mounting medium (Beyotime, Shanghai, China), and a coverslip was placed over it. RGCs were photographed in each retinal quadrant from three equidistant frame samples in each retinal quadrant, with center, medium, and periphery, respectively. Cells in the retina were observed and photographed under a CLSM (ZEISS LSM 880, Germany). RGCs were counted and measured using ImageJ 1.52i software.

**Flow cytometry.** R28 cells were seeded in the six-well plate at a density of 1×10^6^ cells/well overnight. Then, the cells were treated with Nico (10 μM), OA (1 μM), N+O (10 μM) and HOLN-NPs (500 μM) for 7 h. After R28^Glu^ modeling for 24 h, the cells were processed into a single-cell suspension and stained with Annexin V/PI, DCFH-DA, and JC-1 according to the manufacturer's instructions. The above samples were all washed with PBS twice after drying for fluorescence quantitative detection. The data were analyzed with CytExpert software.

**Antioxidant activity *in vitro* and *in vivo*.** Intracellular ROS detection: the cells were seeded at a density of 1×10^5^ cells/well in a 24-well plate. After R28^Glu^ modeling for 24 h, DCFH-DA was diluted 1:1000 in serum-free medium to a final concentration of 10 μmol/L, which was used to incubate R28 cells at 37°C for 30 min in the dark. Next, R28 cells were washed three times to sufficiently remove DCFH-DA that did not enter the cells. The samples were mounted and photographed with a CLSM (ZEISS LSM 880, Germany).

ROS in animal tissues: The retinas of each group were collected in a serum-free medium. After the tissues were homogenized at 4 °C, the DCFH-DA probe was then added to the tissue samples and incubated at 37 °C for 30 min in the dark. The samples were then washed three times, and then resuspended with PBS. The absorbance was read using a Bio-Rad microplate reader (excitation wavelength: 488 nm, emission wavelength: 525 nm).

**NMDA animal model construction.** C57BL/6 mice (8 weeks old; Slaccas, Changsha, China) were fed with standard laboratory food and water in a comfortable environment with a 12 h light–dark cycle. All the experimental procedures were approved by the Institutional Animal Care and Use Committee (IACUC) of Central South University (Changsha, China). All mice were divided into five groups: NMDA, and Nico, OA, N+O, HOLN-NPs. Nico, OA, N+O, and HOLN-NPs were injected into the vitreous 48 h before model construction. All the mice were anesthetized with pentobarbital (1%, 80 mg/kg, intraperitoneal injection; Beijing Sanshu, China) and then operated on under a stereomicroscope. Oxybuprocaine hydrochloride (Santen Pharmaceuticals, Tokyo, Japan) was used to induce ocular surface anesthesia, and tropicamide phenylephrine (Santen Pharmaceuticals) was used to dilate the pupils. A 30-G needle was inserted into the vitreous cavity along the limbus and injected at a volume of 1.5 µL per eye. Tobramycin dexamethasone eye ointment (Alcon Inc, Geneva, Switzerland) was used to prevent infection after injection. The mice were euthanized 7 days after the injection, and their eyeballs were removed with tweezers for the follow-up research.

**Optic Nerve Crush.** All the mice were anesthetized with pentobarbital (1%, 80 mg/kg, intraperitoneal injection; Beijing Sanshu, China) and then operated on under a stereomicroscope. For optic nerve crush, the optic nerve was exposed and crushed intraorbitally with jeweler’s forceps for 5 s, approximately 1 mm behind the globe. Ophthalmic ointment was applied to protect the cornea after surgery.

**IR animal model construction.** C57BL/6 mice (8 weeks old; Slaccas, Changsha, China) were fed with standard laboratory food and water in a comfortable environment with a 12 h light–dark cycle. The retinal ischemia and reperfusion (I/R) model were established following a standard procedure. After the anterior chamber of mice was punctured with a 30 G needle, saline was instilled at a constant speed with a pressure difference of 120 mmHg for 40 min. All animal experiments were reviewed and approved by the Animal Care and Use Committee of the Laboratory Animal Research Center at the Xiangya Medical School of Central South University.

**Hematoxylin-eosin (HE) staining.** After NMDA modeling for 48 h, paraffin-embedded retinal tissue sections (6 µm) were cut in the vertical meridian through the optic disc parallel to the maximum circumference of the eyeball. The sections were mounted onto microscope slides, deparaffinized, and stained with hematoxylin and eosin. Micrographs of stained retinas were generated using an inverted fluorescence microscope (Olympus IX 83, Japan).

**Visual pathway CTB tracing.** For visual pathway CTB tracing, 1.5 ml of Cholera Toxin Subunit B (CTB, Alexa Fluor 555 Conjugate, Thermo Fisher Scientific, C34776) (2 mg/ml in PBS) was injected into the vitreous. Three days after CTB injection, animals were perfused with 4% PFA. Optic nerves and retinas were dissected, fixed, and mounted for imaging. Confocal images were acquired using a Zeiss LSM 880 microscope.

**Western blotting.** The protein expression of CaMKII, p-CaMKII, Cyt C, γ-H2AX, and GAPDH were detected using western blot. Nico, OA, N+O, and HOLN-NPs were injected into the vitreous 48 h before model construction. After NMDA modeling for 48 h, mice were sacrificed and retinal tissues were obtained. The cell lysates (RIPA: cocktail inhibitor = 100:1) were added to retinal tissues. Subsequently, the tissue homogenization and centrifugation were performed. Subsequently, the supernatant was collected and the loading buffer was added. Each group was loaded with 20 μg of the protein sample, and the protein samples were separated by SDS-PAGE gel electrophoresis. Next, the protein was transferred to a PVDF membrane, blocked with 5% milk or 5% BSA for 2 h. Subsequently, antibody diluent was used to mix CaMKII (1:1000), p-CaMKII (1:1000), Cyt C (1:1000), γ-H2AX (1:1000), and GAPDH (1:1000) primary antibodies and incubated at 4°C overnight. The following day, after the PVDF membrane was washed, the [Goat Anti-Rabbit IgG H&L](https://www.abcam.cn/goat-rabbit-igg-hl-hrp-ab6721.html) (1:2000), and [Goat Anti-Mouse IgG H&L](https://www.abcam.cn/goat-mouse-igg-hl-hrp-ab6789.html) (1:2000) were incubated and visualized using a chemiluminescence image analysis system (Tanon 5200, Shanghai, China).

**Electrophysiological examination.** After the mice were anesthetized with 1% pentobarbital sodium, they were placed on a table, and three recording electrodes were inserted separately under the skin of the anterior bregma (cathode), occipital bone (anode), and ear (ground electrode). Unilateral flash VEP data were obtained from the right and left eye in a dark room. When the experiment was complete, the mice were placed in a warm cage for recovery. A professional technician performed the data recording process in a blinded manner. VEP was performed using the Roland ophthalmic electrophysiological diagnostic system (Germany).

**Visual cliff test.** The visual cliff apparatus was purchased from Conduct Science (Boston, MA). The Visual cliff test apparatus consists of a clear plexiglass box, with a dimension of 62 × 62 × 19 cm, separated by a center platform (1.5 inches high and 2 inches wide) into two regions, the shallow side with a checkered pattern immediately under it, and the deep side with a same checkered pattern placed 2 feet under it to create the illusion of depth.^[1,2]^ Mice were placed onto the center platform, and their choices to step down were recorded. Each mouse was subjected to the test once. The box and central platform were thoroughly cleaned after each test.

**Looming visual stimulus response test.** The test for looming visual stimulus response was conducted in an enclosure with dimensions of 17 x 20 x 12 inches, built with materials purchased from 80/20 Inc. (Columbia City, IN) as described.^[3]^ A 5-inch wide board was placed at one end of the enclosure at the height of 3 inches to act as a hideout. Food pieces were placed at the side opposite the hideout to encourage mice to explore their environment and remain outside of the hideout. A monitor was placed on top of the enclosure to display the looming stimulus, a video of an expanding black disk on a gray background made using Blender software. The stimulus parameters were adapted from a previous study ^[4]^, consisting of a circle expanding from a radius of 2-degrees to 20-degrees in 250 ms, where it remained for 250 ms. The stimulus was displayed 15 times, with a 500-ms interval between presentations. An overhead camera recorded mouse behavior. Mice were placed in the enclosure for 10 min prior to stimulus onset to allow time to acclimate. Three responses were assessed during the looming stimulus: freezing, fleeing, and tail rattling. ^[3-4,5-6]^ If a mouse demonstrated at least one of these behaviors throughout of the stimulus, it was tallied as a positive looming responder. Each mouse was subjected to the test once. The enclosure was thoroughly cleaned after each test.

**Statistics.** The final results were obtained from at least three independent experiments, and the variables are presented as mean ± standard deviation. Comparisons of two samples were performed using the student’s t-test, and multiple comparisons were analyzed using a one-way analysis of variance and the Bonferroni’s multiple comparison test. All statistical analyses were performed using GraphPad Prism version 8.0 (GraphPad Software, La Jolla, CA, USA). Statistical significance was set at *p* < 0.05. Asterisks indicate significance on the graphs.

**Figure S1.** Synthetic route of compound **2** (OLN monomer) (A) and **P1** (B).

**Figure S2**. Characterization of compound **1** in DMSO-*d*_6_ by ^1^H NMR.

**Figure S3**. Characterization of compound **2** in DMSO-*d*_6_ by ^1^H NMR.

**Figure S4.** Characterization of compound **3** in DMSO-*d*_6_ by ^1^H NMR.

**Figure S5.** Characterization of compound **4** in DMSO-*d*_6_ by ^1^H NMR.

**Figure S6**. The PDI of HOLN-NPs, HOLN-NPs+H_2_O_2_ and HOLN-NPs+Na_2_S_2_O_4_.


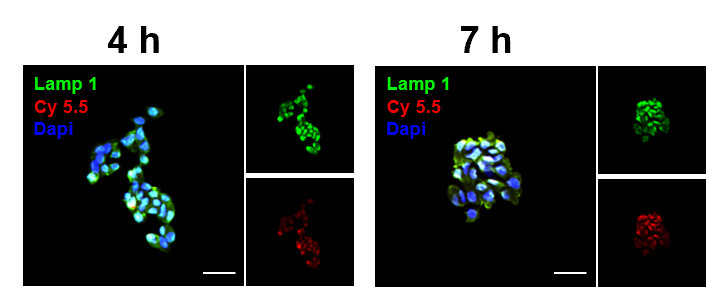


**Figure S7.** Representative CLSM images of R28 cells treated with [HOLN-NPs@Cy5.5](mailto:HOLN-NPs@Cy5.5) at 4 h, and 7 h, respectively. The cell nucleus was stained by DAPI (blue). The red fluorescence came from Cy5.5 (red). The Lysosome was stained by Lamp1 (green) respectively. Scale bar = 30 μm.


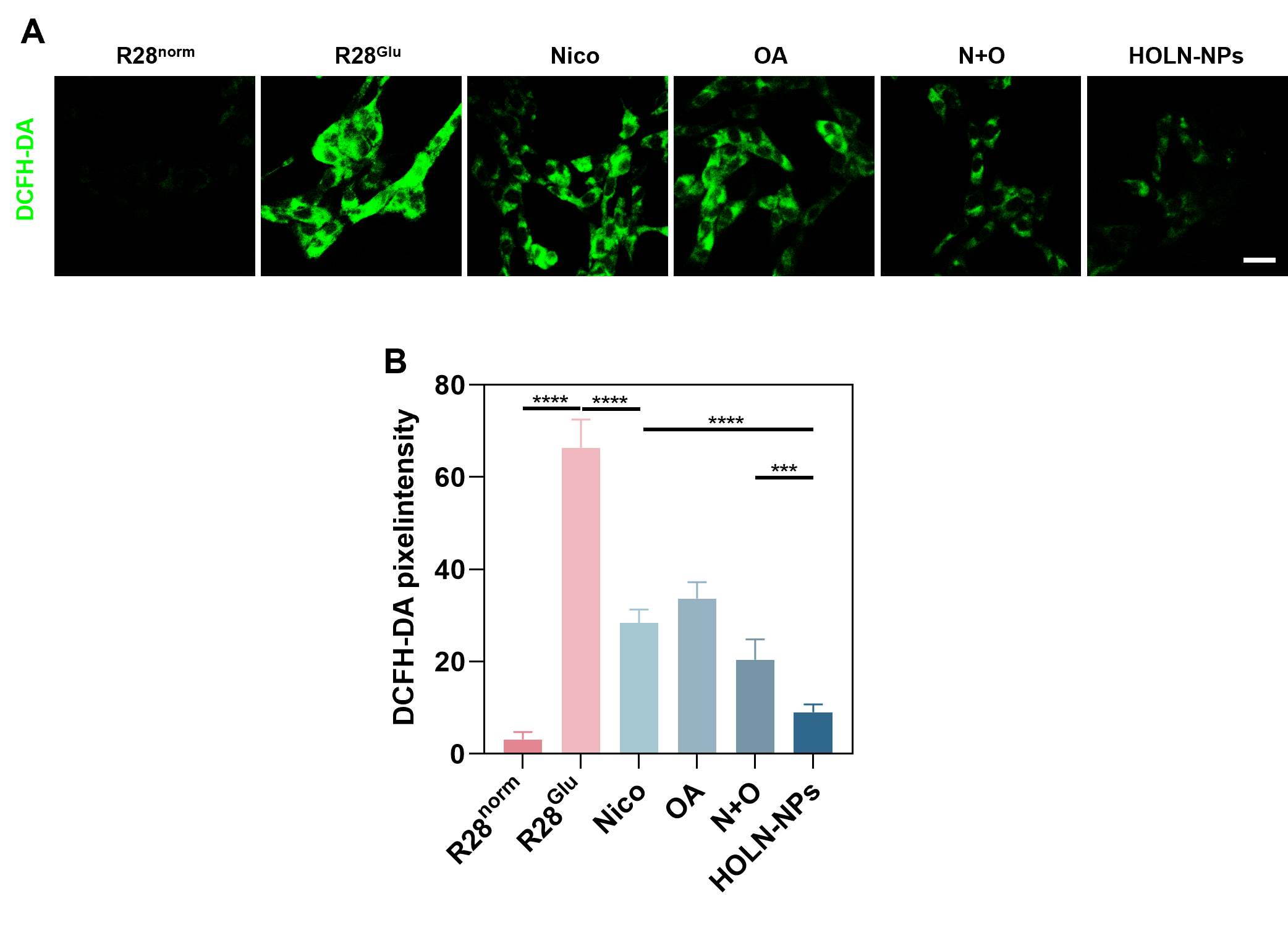


**Figure S8.** A. CLSM images of R28 cells treated with glutamate, Nico, OA, N+O, and HOLN-NPs and incubated with ROS detection probe DCFH-DA. B. The relative pixel intensity of DCFH-DA in R28^Glu^ with various treatments. Scale bar = 20 μm. ****p* < 0.001, and *****p* < 0.0001.


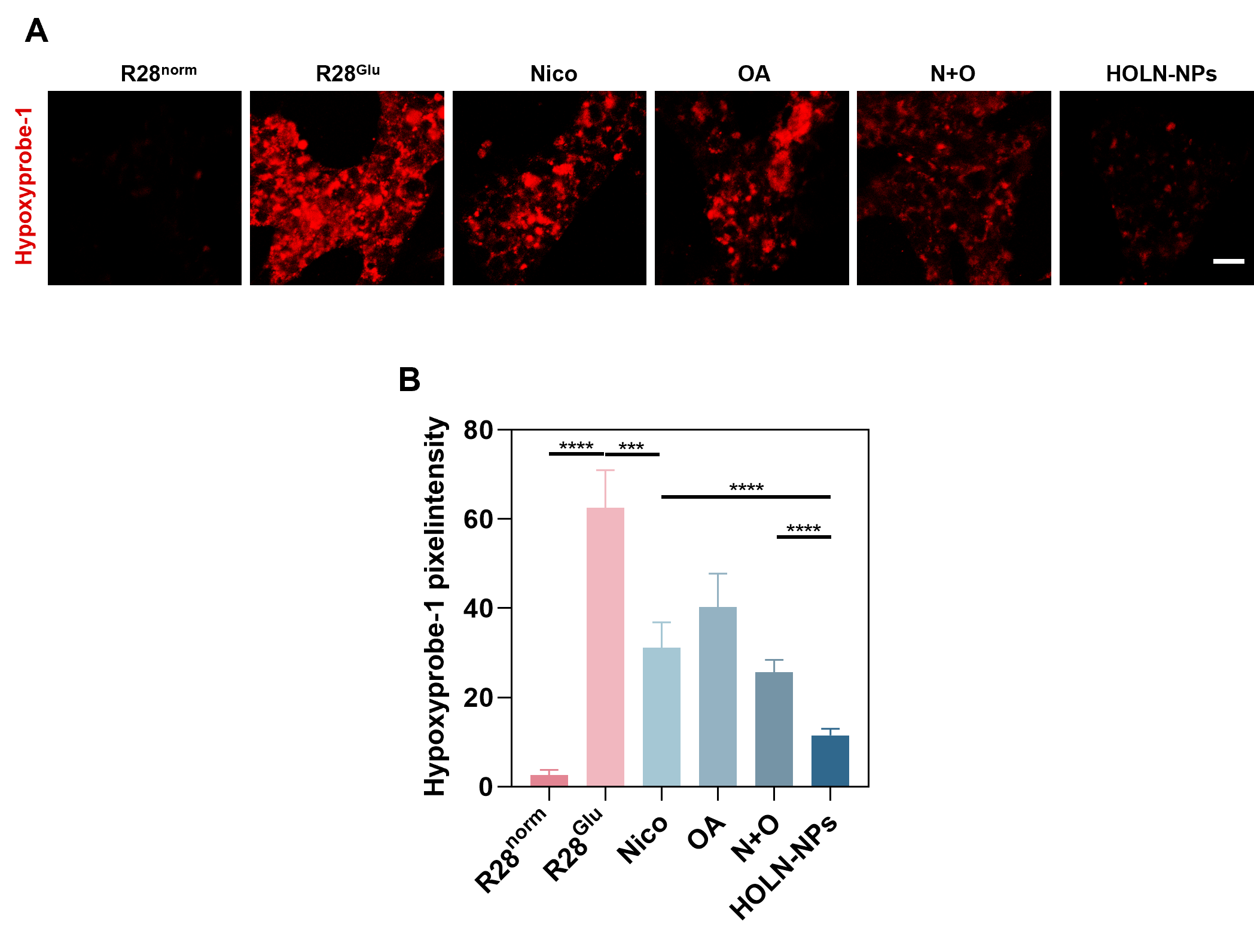


**Figure S9.** A. CLSM images of R28 cells treated with glutamate, Nico, OA, N+O, and HOLN-NPs and incubated with hypoxia detection probe Hypoxyprobe-1. B. The relative pixel intensity of Hypoxyprobe-1 in R28^Glu^ with various treatments. Scale bar = 20 μm. ****p* < 0.001, and *****p* < 0.0001.

**Figure S10.** Relative cell viabilities of R28^Glu^ cells treated various Nico concentration via a CCK-8 assay.

**Figure S11.** Relative cell viabilities of R28 cells treated various Nico concentration via a CCK-8 assay.

**Figure S12.** Relative cell viabilities of R28^Glu^ cells treated various OA concentration via a CCK-8 assay.

**Figure S13.** Relative cell viabilities of R28 cells treated various OA concentration via a CCK-8 assay.


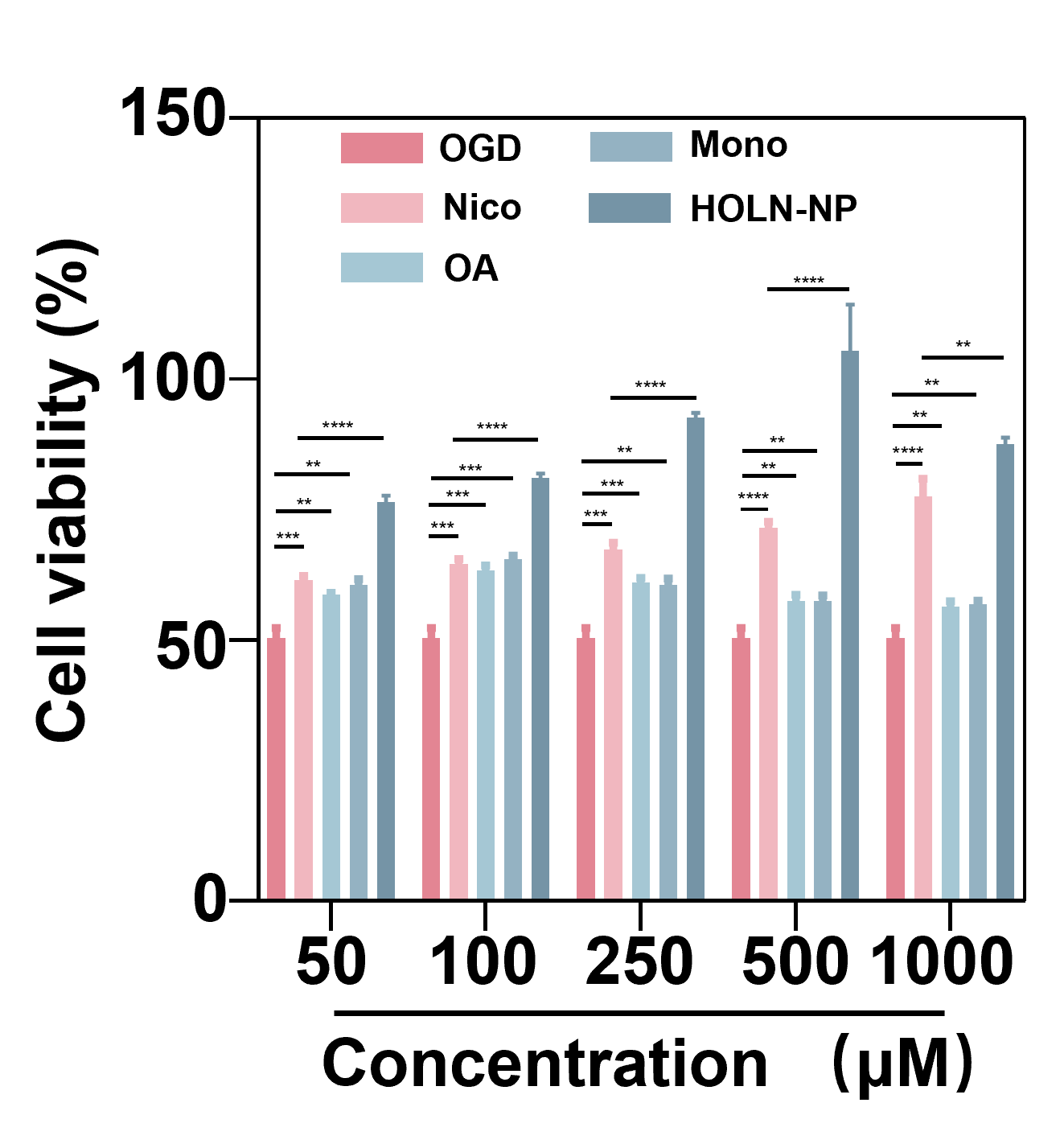


**Figure S14.** *In vitro*, cell viability of R28^OGD^ cells treated by Nico, OA, monomer, and HOLN-NPs at different concentrations by CCK-8. ***p* < 0.01, ****p* < 0.001, and *****p* < 0.0001.


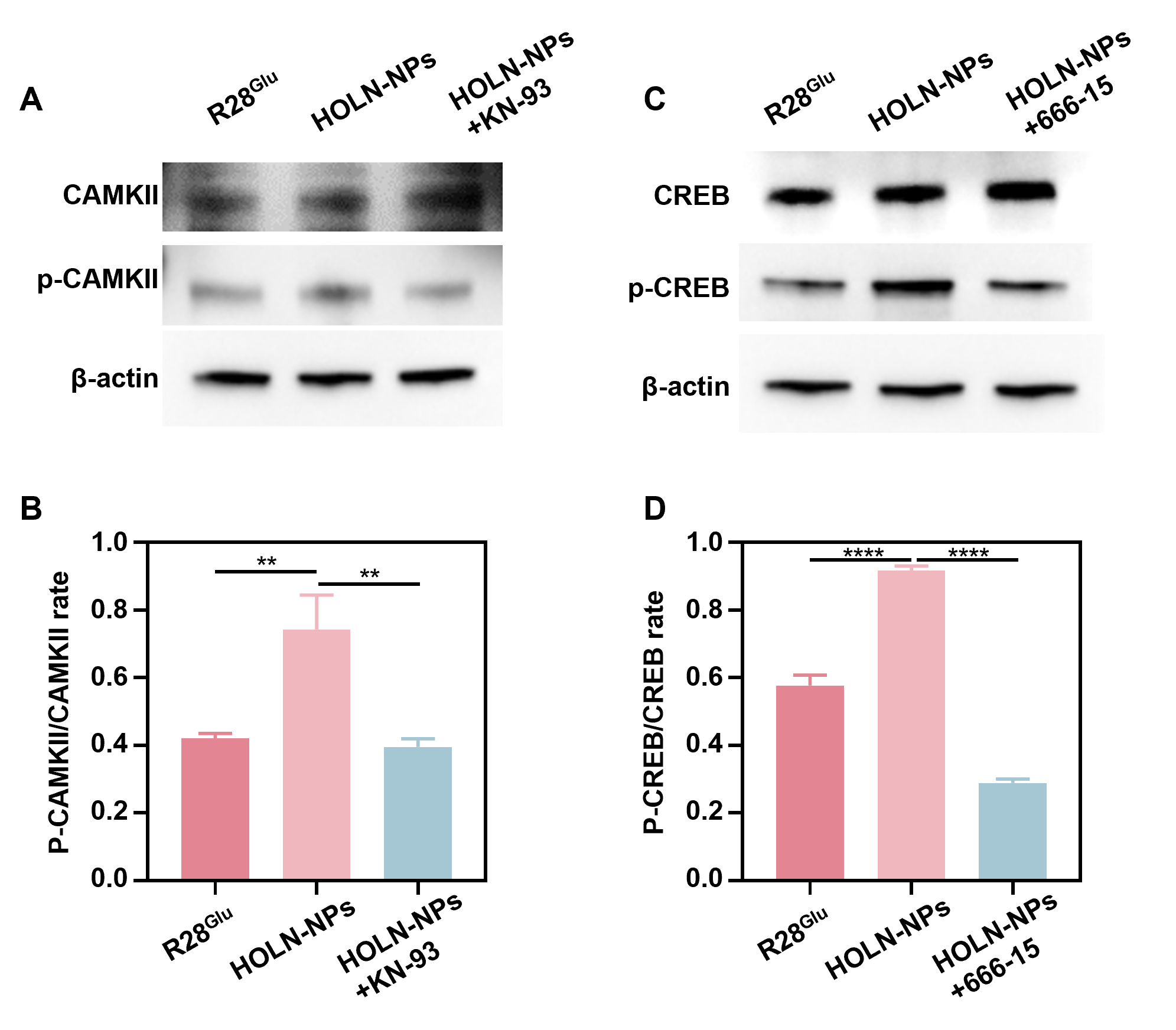


**Figure S15.** The expression level of p-CaMKII and p-CREB in the R28 with different treatments by WB. Statistical significance was calculated via one-way ANOVA analysis. ***p* < 0.01 and *****p* < 0.0001.

**Figure S16**. Relative cell viabilities of R28 cells treated with Glu, HOLN-NPs, KN-93 and 666-15 via a CCK-8 assay. Statistical significance was calculated via one-way ANOVA analysis. ***p* < 0.01 and ****p* < 0.001.


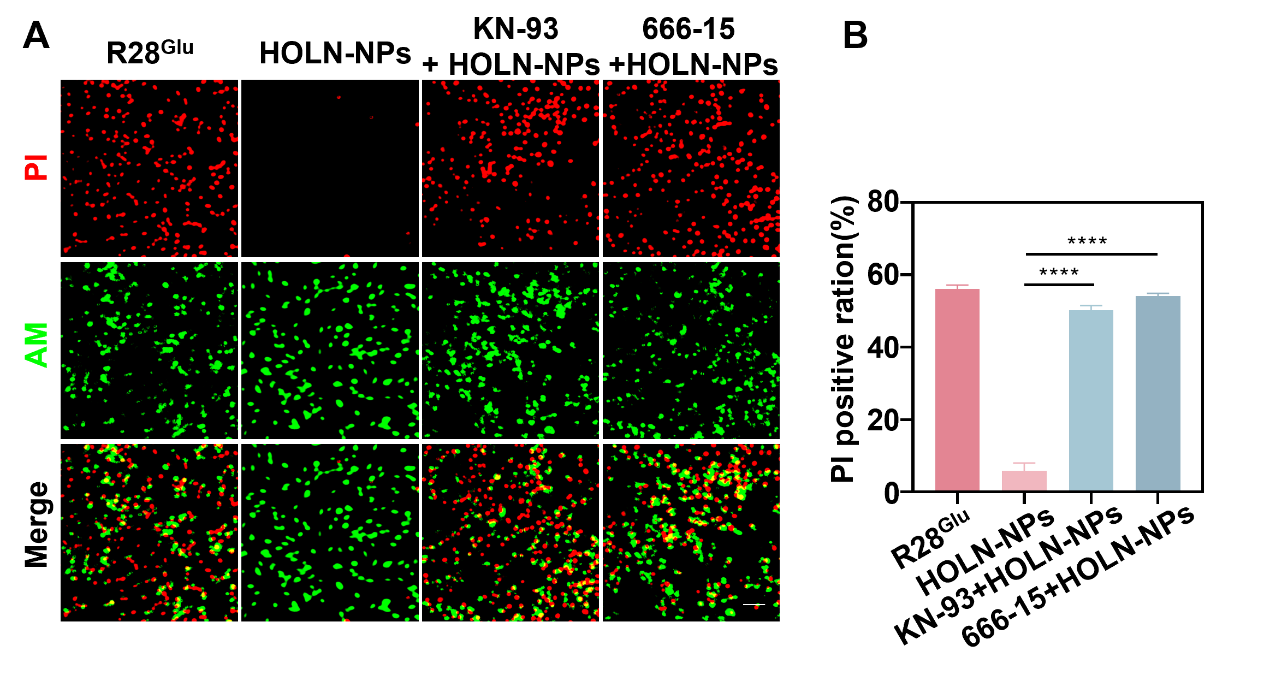


**Figure S17.** The representative CLSM images of R28Glu were stained with Calcein-AM (green) and PI (red) after treatment with HOLN-NPs, KN-93 + HOLN-NPs and 666-15 + HOLN-NPs for 24 h. Scale bar=50 μm. Data are presented as the mean ± SD (n = 3). Statistical significance was calculated via one-way ANOVA analysis. *****p* < 0.0001.

**Figure S18.** Intracellular uptake of HOLN-NP@Cy5.5 by retina RGCs at Day 1, Day 2 and Day 7 by CLSM. Scale bar=100 μm.


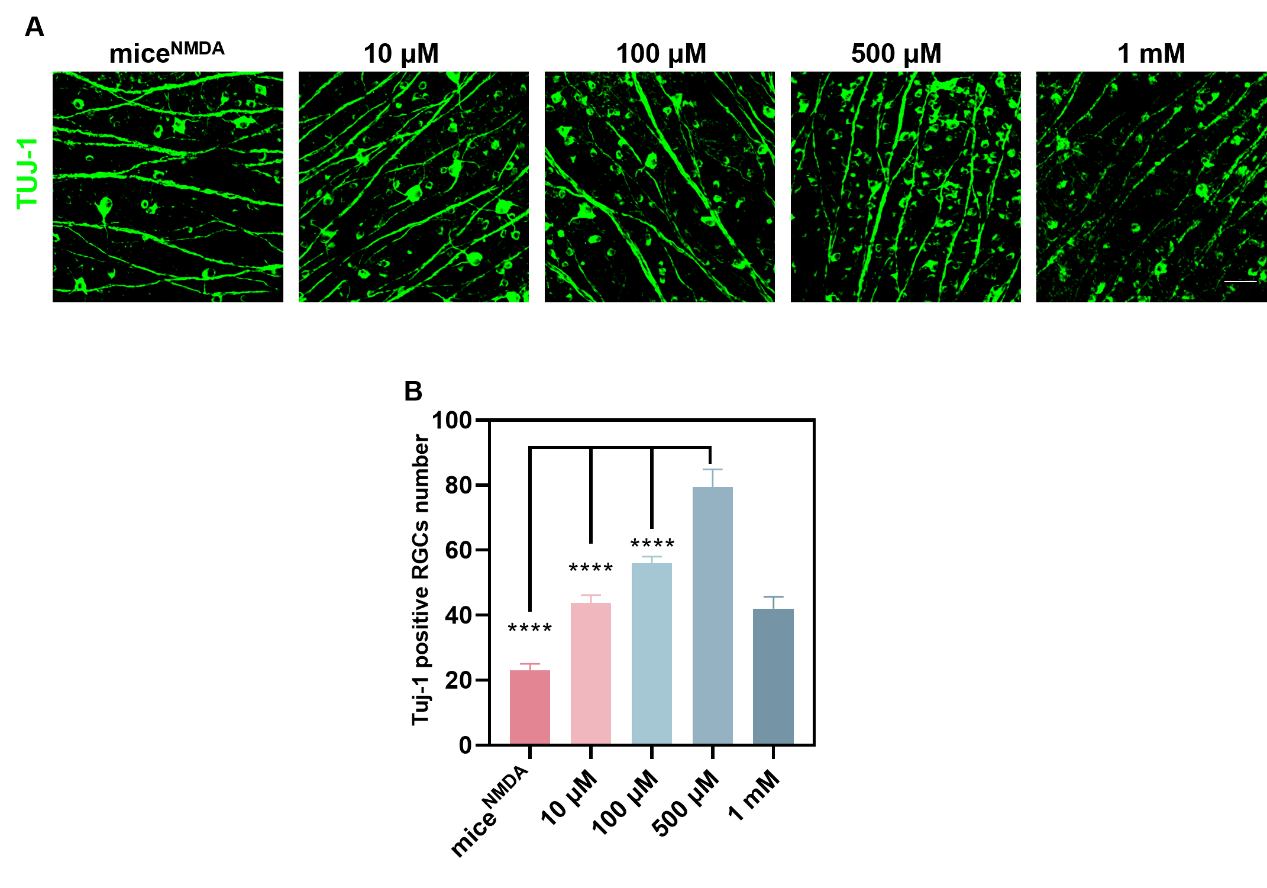


**Figure S19.** CLSM images of TUJ-1 labeled RGCs of mice^NMDA^ retinas treated with different concentrations of HOLN-NPs. Scale bar = 20 μm. Statistical significance was calculated via one-way ANOVA analysis. *****p* < 0.0001.


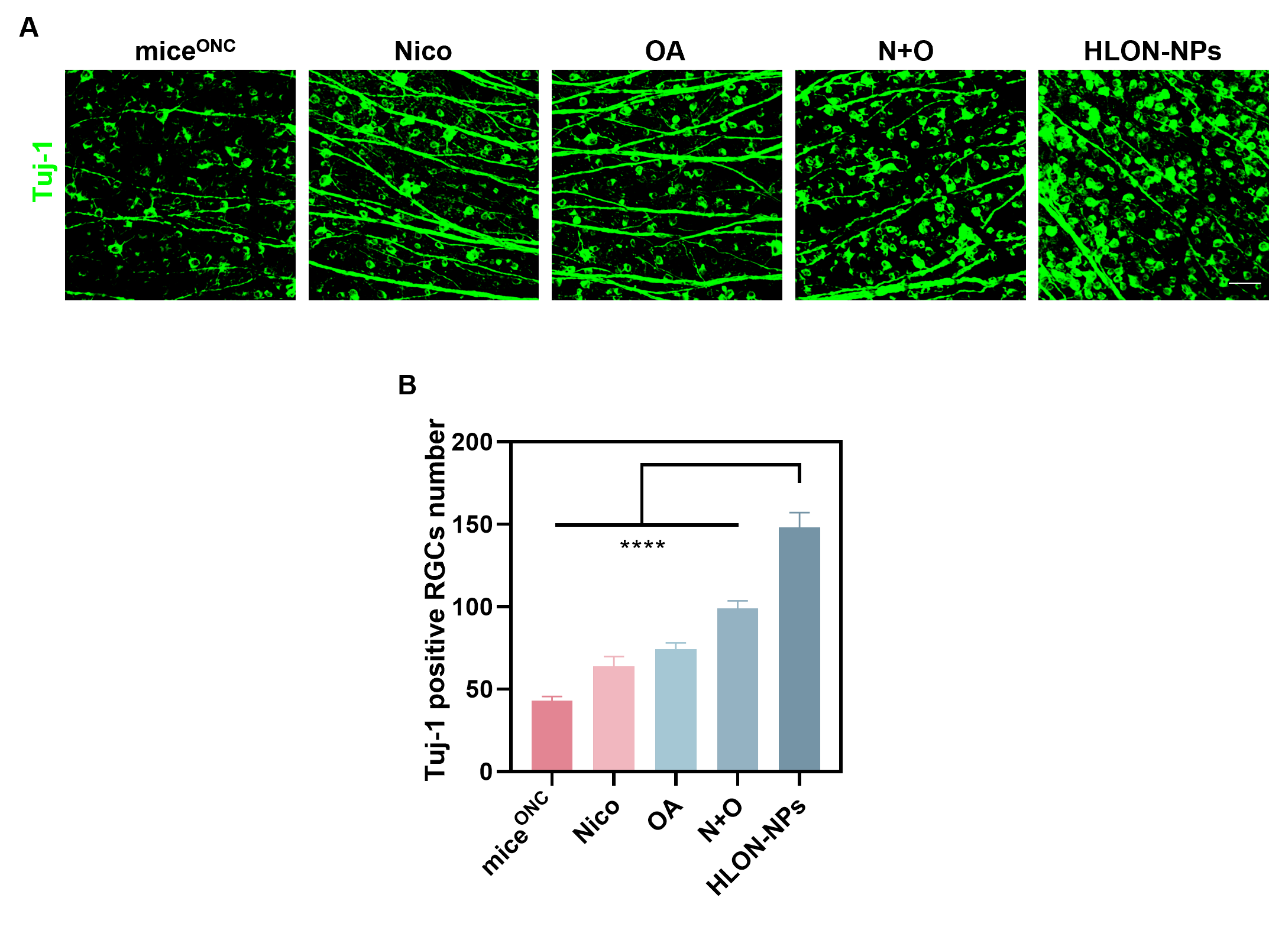


**Figure S20**. CLSM images of TUJ-1 labeled RGCs of mice^ONC^ retinas treated with Nico, OA, N+O, and HOLN-NPs. Scale bar = 20 μm. Statistical significance was calculated via one-way ANOVA analysis. *****p* < 0.0001.

**Figure S21.** CLSM images of mice^ONC^ retinal whole mounts showing p-CaMKII in TUJ-1-labeled RGCs at 12 h after treating with Nico, OA, N+O and HOLN-NPs. Scale bar=20 μm.


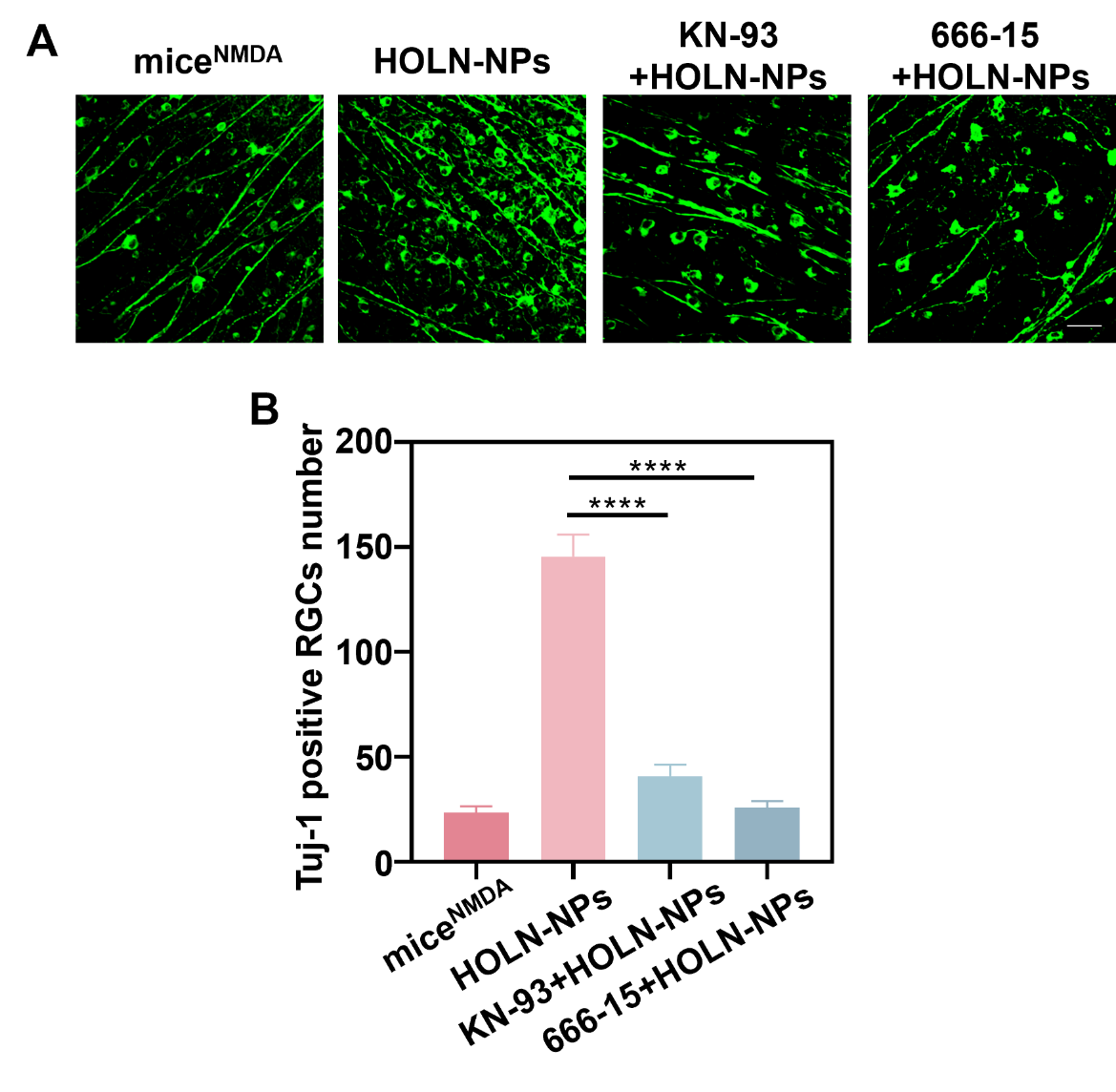


**Figure S22.** TUJ-1 labeled RGCs in flattening of mice^NMDA^ retina treated with HOLN-NPs, KN-93 + HOLN-NPs, and 666-15 + HOLN-NPs. Scale bar = 20 μm. Statistical significance was calculated via one-way ANOVA analysis. *****p* < 0.0001.


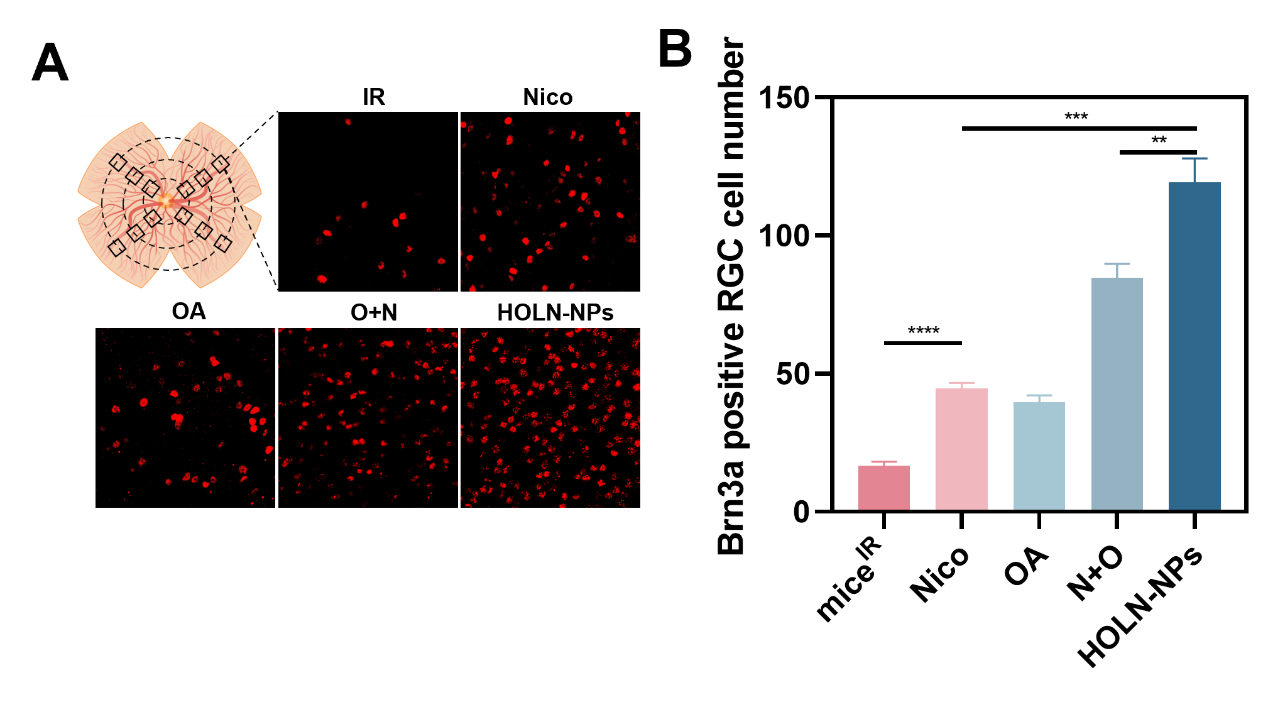


**Figure S22**. CLSM images of Brn3a labeled RGCs of mice^IR^ retinas treated with Nico, OA, N+O, and HOLN-NPs. ***p* < 0.01, ****p* < 0.001, and *****p* < 0.0001.


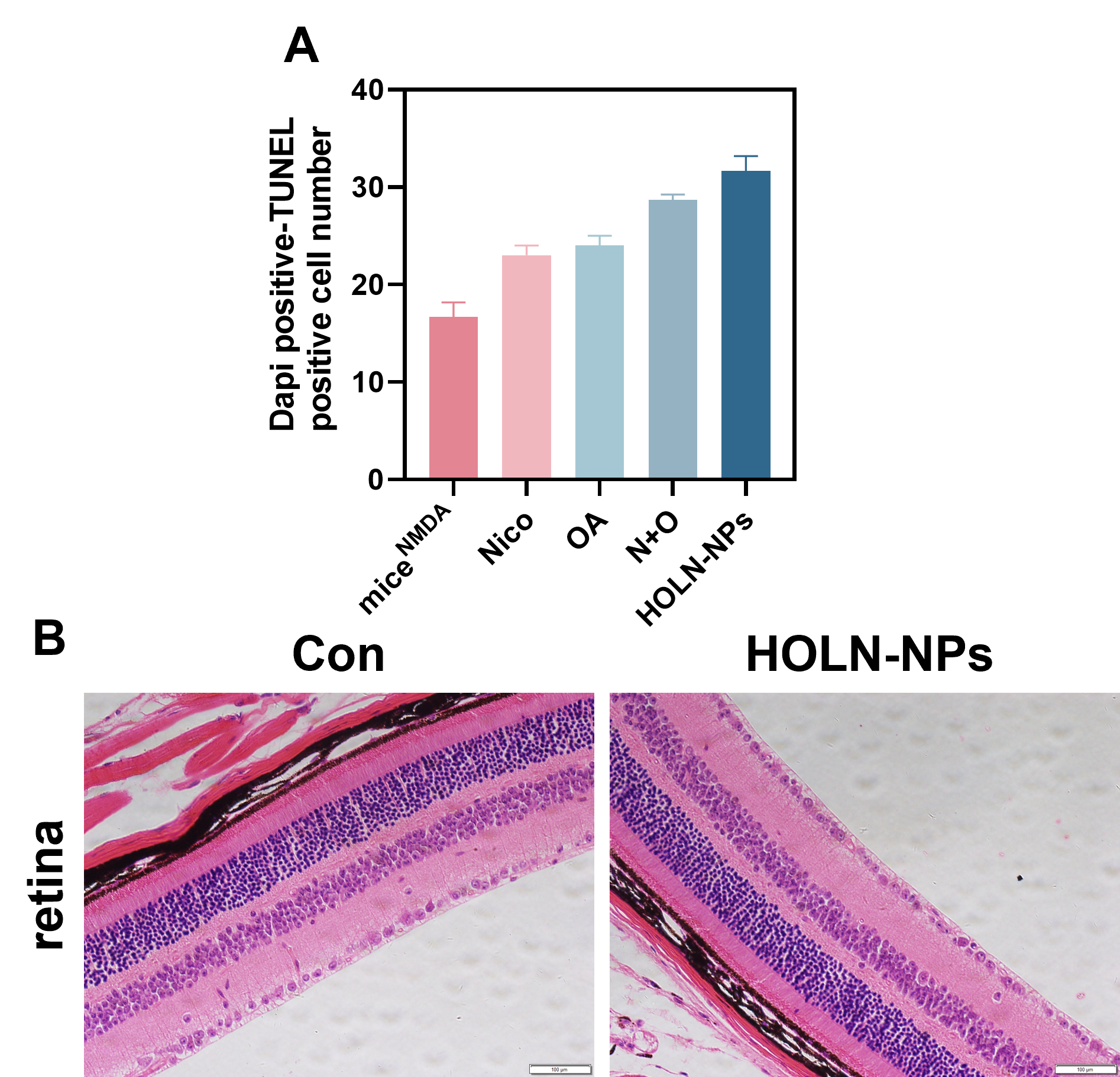


**Figure S24.** A. The number of viable retinal RGCs cells after TUNEL staining. B. H&E staining of retina tissues in normal group and HOLN-NP intraocular injection group. Scale bar = 100 μM.


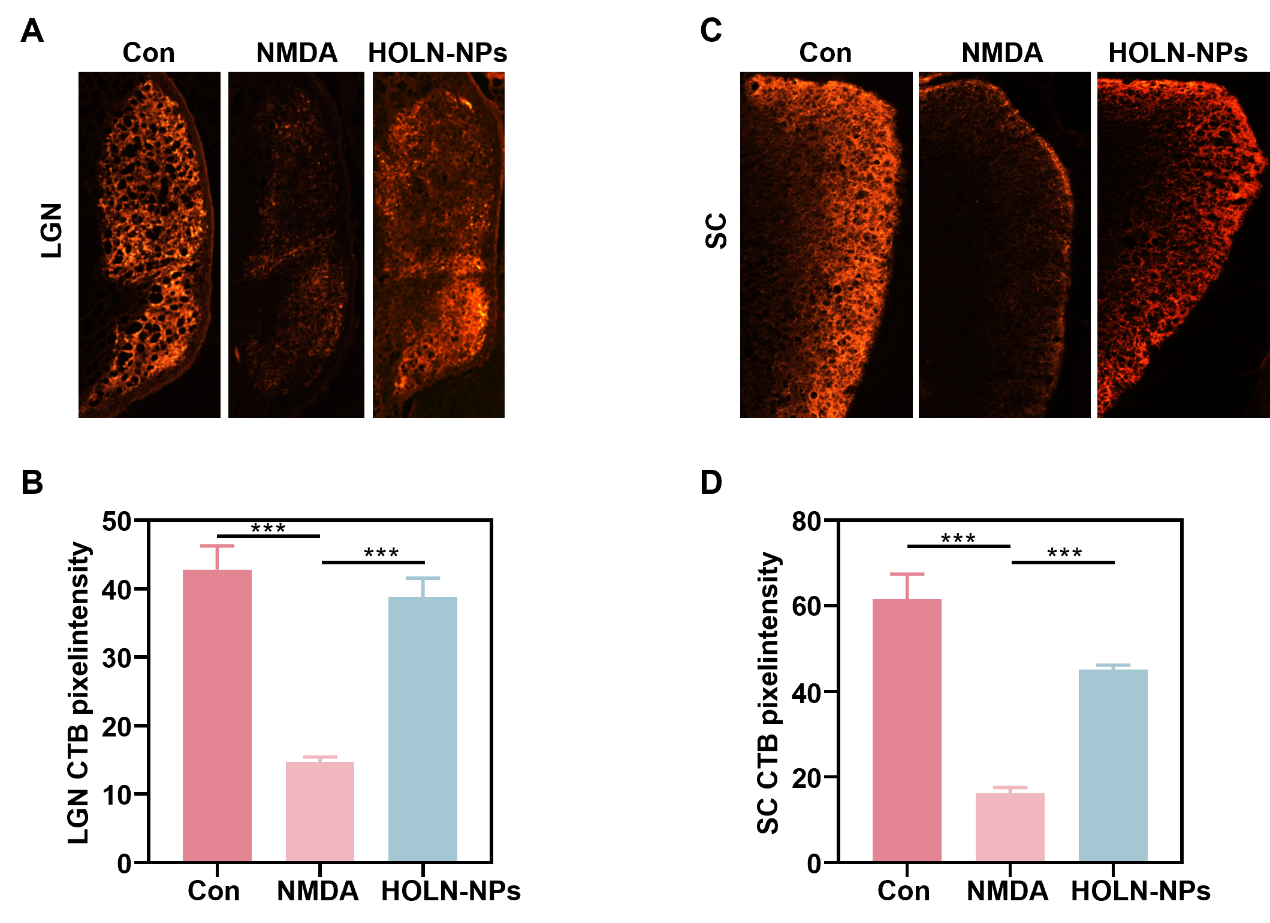


**Figure S25.** The CLSM images of CTB labeled LGN (A-B) and SC (C-D) in different groups. Statistical significance was calculated via one-way ANOVA analysis. ****p* < 0.001.

**Figure S26.** The F-ERG results of mice^ONC^ eyes after different treatments.

**Figure S27.** (A-B) Principal component analysis of all detectable metabolites in mice^NMDA^ treated with PBS and HOLN-NPs.

**Figure S28.** Pearson correlation analysis heat map of retinal metabolites in mice^NMDA^ and HOLN-NPs treatment groups.


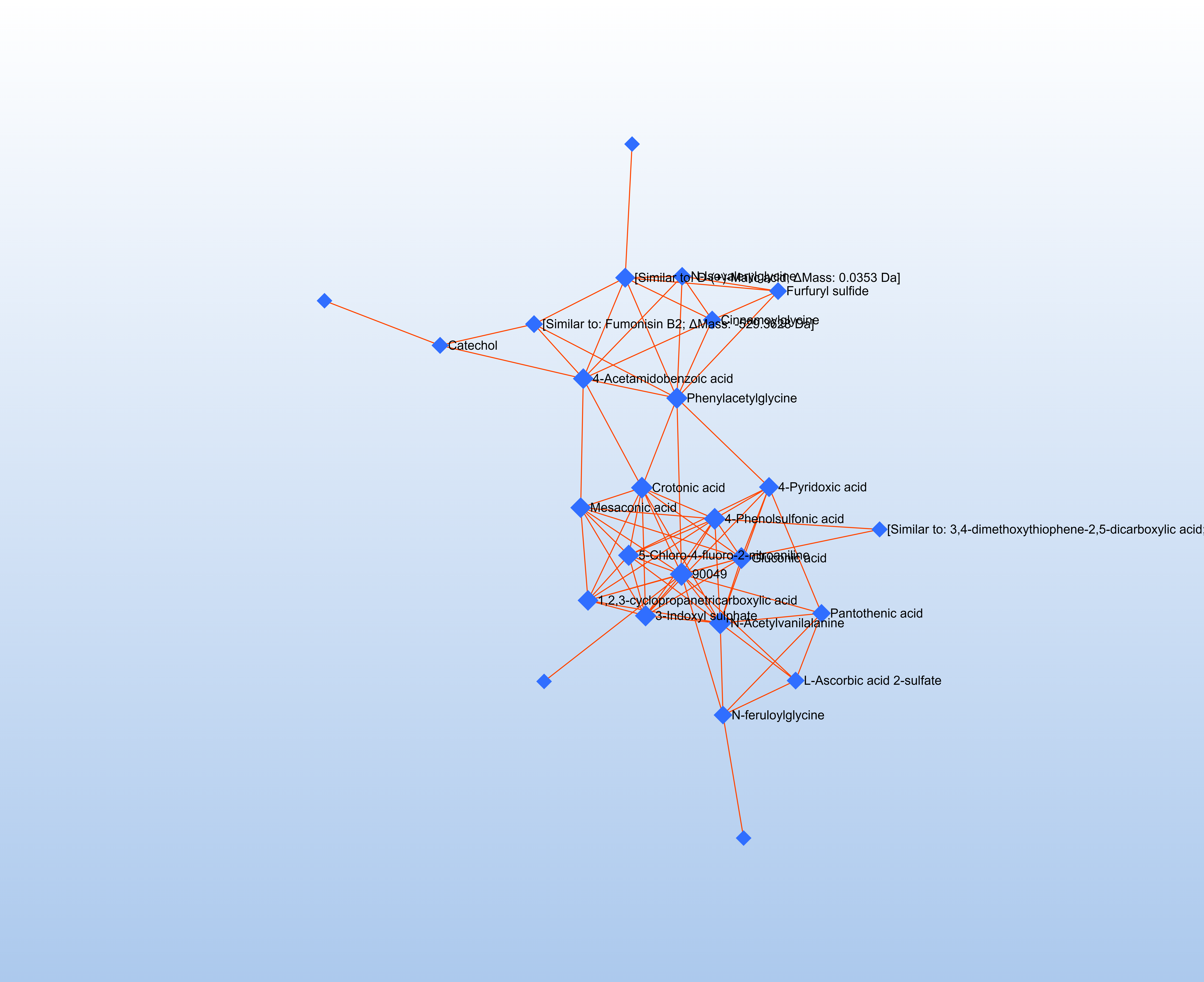


**Figure S29.** High definition enlarged image of Figure 8L.

**Reference**

1. Fox MW. The visual cliff test for the study of visual depth perception in the mouse. Anim Behav. 1965 Apr-Jul;13(2):232-3.
2. Gu L, Bok D, Yu F, Caprioli J, Piri N. Downregulation of splicing regulator RBFOX1 compromises visual depth perception. PLoS One.
3. Koehler CC, Hall LM, Hellmer CB, Ichinose T. Using Looming Visual Stimuli to Evaluate Mouse Vision. J Vis Exp. 2019 Jun 13;(148):10.3791/59766.
4. Yilmaz M, Meister M. Rapid innate defensive responses of mice to looming visual stimuli. Curr Biol. 2013 Oct 21;23(20):2011-5.
5. Lim JH, Stafford BK, Nguyen PL, Lien BV, Wang C, Zukor K, He Z, Huberman AD. Neural activity promotes long-distance, target-specific regeneration of adult retinal axons. Nat Neurosci. 2016 Aug;19(8):1073-84.
6. Salay LD, Ishiko N, Huberman AD. A midline thalamic circuit determines reactions to visual threat. Nature. 2018 May;557(7704):183-189.
